# Protocol of a randomised controlled multicentre trial investigating the effectiveness and safety of a wilderness programme on the mental and physical well-being of adolescents and young adults affected by cancer: the WAYA-2 study

**DOI:** 10.1136/bmjopen-2024-087626

**Published:** 2024-05-21

**Authors:** Miek C Jong, Carina Ribe Fernee, Andreas Stenling, E Anne Lown, Sveinung Berntsen, David E Victorson, Mats Jong

**Affiliations:** 1 Department of Health Sciences, Mid Sweden University, Sundsvall, Sweden; 2 Department of Community Medicine, National Research Center in Complementary and Alternative Medicine (NAFKAM), UiT The Arctic University of Norway Faculty of Health Sciences, Tromso, Norway; 3 Department of Child and Adolescent Mental Health, Sorlandet Hospital Kristiansand, Kristiansand, Norway; 4 Department of Sport Science and Physical Education, University of Agder, Kristiansand, Norway; 5 Department of Psychology, Umeå University, Umea, Sweden; 6 Department of Social & Behavioral Sciences, University of California, San Francisco, California, USA; 7 Department of Medical Social Sciences, Feinberg School of Medicine, Northwestern University, Chicago, Illinois, USA

**Keywords:** safety, quality of life, oncology, psychosocial intervention, self-management

## Abstract

**Introduction:**

Adolescents and young adults (AYAs) affected by cancer are an understudied group. Effective interventions are needed to support coping with the late effects of cancer, its treatment and to promote quality of life. Nature-based interventions may be promising in support of the self-management and health of AYAs affected by cancer. However, randomised controlled studies (RCTs) on the effectiveness of such interventions are lacking. We performed a first pilot RCT (n=42) that showed that it is feasible and safe to conduct such a study. Here, we propose a full-scale RCT to investigate the effectiveness and safety of a wilderness programme on the mental and physical health of AYAs affected by cancer.

**Methods and analysis:**

Participants are 150 AYAs affected by cancer, aged 16–39 years, who will be randomised to a wilderness (n=75) or a hotel stay (n=75). The wilderness programme is an 8-day intervention including a 6-day wilderness expedition. This is followed 3 months later by a 4-day intervention including a 2-day basecamp. Activities include hiking, backpacking, kayaking, rock climbing, mindfulness and bush-crafting. The comparison group is an 8-day hotel stay followed by a 4-day hotel stay (interventions include two travel days) at the same hotel after 3 months. Primary outcomes are psychological well-being and nature connectedness up to 1 year after the study start. Secondary outcomes are quality of life, physical activity and safety parameters.

**Ethics and dissemination:**

The Swedish Ethical Review Authority approved the study protocol on 27 September 2023 (reference: 2023-05247-01). The recruitment started on 19 February 2024 and the first part is planned to end on 31 December 2027. Study results will be disseminated by means of scientific publications, presentations at conferences, popular articles, interviews, chronicles and books. News items will be spread via social media, websites and newsletters.

**Trial registration number:**

ISRCTN93856392.

**STRENGTHS AND LIMITATIONS OF THIS STUDY**

- The feasibility of this randomised controlled trial design has previously been piloted.
- This study will be carried out by a multidisciplinary research team with strong support from patient and public involvement.
- This study aims to investigate the long-term health effects of a wilderness programme.
- This study will include a heterogeneous cancer study population.
- Comparison to no programme participation or a wait-list control will not be included in this randomised controlled trial.

## Introduction

Adolescents and young adults (AYAs) affected by cancer, either by a childhood or AYA cancer diagnosis, are a growing but understudied population.^1^ Due to cancer or its treatment, many AYAs experience at least one late effect such as psychosocial problems, concentration/memory difficulties and fatigue that negatively impact their quality of life.^2^ Furthermore, they suffer disproportionately from chronic diseases and have an excess risk for early mortality compared with non-cancer populations.^3^ It appears that AYAs affected by cancer receive little follow-up care that appropriately addresses these late effects.^2^ Consequently, different self-management strategies have evolved by which they are trying to cope with late effects. These include pushing themselves physically and mentally to master everyday activities, balancing social activities and rest, and recuperating and regaining energy.^4^ Other coping strategies reported are seeking distraction, support from family and friends, yoga or meditation, or changes in lifestyle such as increased physical activity and healthy diets.^5^ Although AYAs affected by cancer acknowledge the importance of these self-management strategies in dealing with the late effects of cancer or the cancer treatment itself, it remains an ongoing challenge.^5^ Therefore, it is a high priority to find effective interventions that support the self-efficacy of AYA impacted by cancer, thereby minimising the negative impact of cancer-related late effects and improving their quality of life.^5^

Physical activity-based interventions have been shown to increase physical activity and reduce fatigue in childhood cancer survivors.^6–8^ However, results must be interpreted with caution due to the small sample sizes and large heterogeneity among the reviewed studies.^7^ Physical activity-based interventions have also been reported to increase the quality of life of AYA cancer survivors, however, with marginal significance.^9^ It appears to be difficult for AYAs affected by cancer to return to or maintain adequate levels of physical activity, and they seem to make little use of existing supporting physical activity services within the community.^10^ A number of barriers exist that make it more difficult to engage in physical activity including self-reported fears about health, low baseline activity levels and/or fitness, lack of motivation, cancer-related pain or fatigue, age at diagnosis, anxiety about health and lack of advice from a physician.^11 12^

Another promising intervention in support of the self-management of cancer survivors is nature-based interventions. Those affected by cancer have reported that engaging with nature is important for their health and well-being.^13–15^ Research from different fields indicates that nature-based interventions may decrease anxiety, depression, stress, tension, fatigue, pain and improve sleep in those affected by cancer.^16^ Nature may provide a safe space and motivational context for patients with cancer that supports relaxation, recovery, physical activity and may ignite reflection, inspiration, personal growth and supportive relationships.^14 17 18^ Furthermore, it has been demonstrated that spending time in nature leads to more frequent or longer stays in nature.^19^

To gain insight into the possible beneficial effects of nature-based interventions specifically for AYAs affected by cancer, we first performed a scoping review on the topic.^20^ Nature-based interventions such as adventure and wilderness programmes were found to increase self-efficacy, self-esteem, self-confidence, physical activity and social support among AYAs affected by cancer. However, it was found that randomised controlled trials (RCTs) on the effectiveness of nature-based interventions were lacking. Other gaps identified were the lack of studies on the long-term effects (>3 months) of nature-based interventions and lack of data on the safety of these interventions.^20^ Therefore, in 2021–2022, we performed a pilot study to investigate whether it would be feasible to perform an RCT design on a nature-based intervention.^21^ For this pilot study, we developed a Wilderness programme for AYAs (WAYA programme) affected by cancer. The WAYA programme is grounded in Næss’s ecosophy^22^ and the Positive Health Model.^23^ A detailed description and schematic outline of the WAYA programme theory and content has previously been published.^24^ We demonstrated that it was feasible and safe to conduct a pilot RCT.^25^ None of the 42 AYAs that participated in the interventions dropped out of the study, and adherence to programme interventions and data completeness after 1-year follow-up was high. The WAYA programme was found to be just as safe for AYAs affected by cancer as a hotel stay. The relative frequency of reported adverse effects (AE) was similar in both study arms (relative risk (RR) 1.0, 95% CI 0.8 to 1.3), and all AEs were non-serious, mild or moderate of severity, and resolved during the study period.^25^ Furthermore, the WAYA programme appeared to be acceptable, developmentally appropriate and of interest to AYA affected by cancer.^24 25^ Based on these observations, we recommended for a future larger RCT to (1) apply additional recruitment strategies for participants, (2) to stratify for the inclusion of cancer survivorship (childhood cancer vs AYA cancer), (3) to optimise the content of the WAYA programme and (4) to select health-related outcomes based on the lived experiences of participants in the WAYA programme.^26^

The aim of this study is to perform a full-scale multicentre RCT (WAYA-2 study) that investigates the effectiveness and safety of a wilderness programme on the mental and physical well-being of AYAs affected by cancer. The WAYA programme and WAYA-2 study protocol have been optimised according to the findings and recommendations from the pilot study as described above.

## Methods and analysis

### Study guidance and design

The WAYA-2 study protocol is developed in line with the Standard Protocol Items: Recommendations for Interventional Trials (SPIRIT) 2013 guidance for protocols of clinical trials and the SPIRIT 33-item checklist.^27^ The WAYA-2 study will be conducted in accordance with the guidelines of the Declaration of Helsinki, and results will be reported according to the Consolidated Standards of Reporting Trials, 25-item checklist and flow diagram.^28^ This is a multicentre RCT in Norway and Sweden with a two-armed parallel design using individual randomisation. A quantitative RCT design will allow the testing of active aspects of a complex wilderness intervention including physical activity, physical challenges, experiential activities, being in nature and other reflective practices. A hotel stay is chosen as a comparison to control for other possible effective factors such as attention, group support and getting out of their regular environment.

### Research questions

The following research questions will be addressed:

Does a wilderness programme, including a wilderness expedition and basecamp stay:

1. Increase psychological well-being of AYAs affected by cancer compared with a control activity.
2. Increase nature connectedness of AYAs affected by cancer compared with a control activity.
3. Improve health-related quality of life of AYAs affected by cancer compared with a control activity.
4. Increase physical activity level of AYAs affected by cancer compared with a control activity.
5. Is a wilderness programme equally safe for AYAs affected by cancer as a hotel stay (control activity)?

### Participants and inclusion criteria

Participants are adolescents and young adults aged 16–39 years that have been diagnosed with any type of cancer during childhood, adolescence or young adulthood. Another inclusion criterion is the ability to walk 2 km a day (walking aids or assistance from another person is permitted). Participants with various disabilities such as mobility, vision and hearing impairments, balance problems and special treatment or dietary needs can be included. Prior experience with outdoor activities is not required for study participation.

Exclusion criteria are as follows:

- Cancer treatment imposes unwanted risks for AYAs on programme participation, as evaluated by their treating physician/oncologist.
- A medical condition of AYAs that prevents safe travel to, or participation in the programme, as evaluated by their treating physician/oncologist.
- Not willing to be randomly assigned to programme interventions.
- Previous participation in the WAYA-1 study.

Following the recommendations of AYA cancer experts in our advisory board, we will deliberately refer to participants in the WAYA-2 study as AYAs affected by cancer, rather than childhood and AYA cancer survivors.

### Intervention group: wilderness programme

The development of the WAYA programme affected by cancer has previously been published.^24^ The WAYA programme aims to increase physical activity, self-confidence, self-efficacy and self-care, to support personal growth and building meaningful relationships, and to provide an enjoyable and safe stay in nature. Nature in the WAYA programme has both a contextual and an active role in support of the health and well-being of AYAs affected by cancer, wherein their health is regarded to be intrinsically interwoven in a bidirectional fashion with the health and well-being of nature.^29^ Participants are given the opportunity to experience a diversity of natural landscapes, and specific nature characteristics are actively incorporated in programme activities. Activities in the programme include hiking, sea-kayaking, backpacking, rock climbing and camping. The programme also includes (nature) reflective practices such as mindfulness, meditation and forest bathing.^24^ Outdoor skills taught include mapping/compass/orienting, trail cooking, safety training, equipment planning, foraging, fishing, bush-craft skills and leaving no trace. The wilderness programme consists of three intervention parts: (1) An 8-day intervention that includes 2 days for travelling and physical testing and a 6-day wilderness expedition; (2) A 3-month in-between period where participants are contacted once every month online to coach them to engage in their own outdoor activities and (3) A 4-day intervention including a 2-day base camp programme. Both the expedition and basecamp programme will take place in natural settings around the High Coast of Sweden or in Southern Norway. The group size will be 10–12 participants, and the facilitator–participant ratio will be 3:10. The 6-day wilderness expedition will be logistically supported by a team of 5–7 volunteers.

### Control group: hotel stay

The hotel stay will be for 8 days at a Spa Hotel in Sweden or Norway, including 2 days for travelling and physical testing and a 6-day hotel stay, followed by a second 4-day stay at the same hotel (including two travel days) 3 months later. Similar to the wilderness intervention, during the 3-month in-between period, participants will be contacted once every month to ask how they are doing. The content of the hotel stay includes the following activities: Spa facilities, museum visits, watching television/movies, playing games/gaming, shopping and fine dining. Participants will engage in organised group activities, but this does not include any nature-based activities. The group size is 10–12 participants, and the facilitator–participant ratio will be 3:10.

### Program facilitators

In Sweden and Norway, we will work with professional teams of outdoor instructors and facilitators who have previous experience in wilderness programmes with young cancer survivors or other vulnerable youth populations. Facilitators of the WAYA programme have competence in one or more of the following areas: outdoor life, survival, nature/wilderness guiding, kayaking, climbing, nursing, first aid, (youth/group) counselling/supervising, mindfulness, mind-body techniques and data collection for research purposes.^24^ At least two facilitators of the WAYA programme are licensed health professionals (nurses, psychologists) and at least one facilitator of the WAYA programme and hotel stay is an AYA affected by cancer. For programme intervention fidelity, referring to the degree to which the WAYA programme is performed in a similar manner in Sweden and Norway, two members of the Norwegian outdoor team will join the Swedish outdoor team for the first wilderness intervention in Sweden and vice versa. For supervision of participants in the hotel stay, facilitators will have competence in guiding/supervising groups, first aid and research methodology for data collection and safety monitoring.

### Outcomes

The selection of outcomes is driven by the aims of the WAYA programme and recently published qualitative study results of the WAYA programme.^26^ The aims of the WAYA programme and underlying process to support self-realisation via nature connectedness are hypothesised to contribute to a chain of actions that impacts the six domains of Positive Health.^25^ The WAYA programme is a complex intervention and may potentially impact multiple factors. Therefore, in line with guidance and instructions for RCTs on complex interventions, more than one primary outcome is chosen in relation to the research questions.^30^

#### Primary outcomes (confirmatory)

Changes in psychological well-being of AYAs affected by cancer over time, measured by the Psychological Well-being Scale (PWS).^31^ This scale consists of six domains: self-acceptance, positive relations with others, autonomy, environmental mastery, purpose in life and personal growth. Together, these dimensions can contribute to the assessment of a person’s level of positive functioning and well-being. This instrument is translated and validated in the Swedish context.

Rationale: Significant and relevant changes in the PWS have been demonstrated for complex interventions including mindfulness-based programmes.^32^ The PWS was not included as an outcome measure in our previous pilot study,^1^ but based on the findings from the qualitative study of the WAYA programme, we have selected PWS as a primary outcome measure.^26^ For the qualitative analysis of the pilot study, participants in the wilderness programme were interviewed at 3 months after start of the programme to explore the impact of the programme on their health and well-being. The themes that arose through the inductive content analysis related to self-confidence, self-acceptance, meaningful relationships and personal growth. These themes are also reflected within the domains of the PWS providing additional richness to the data.^31^

Changes in nature connectedness of AYAs affected by cancer over time, measured via the Nature Relatedness Scale (NRS).^33^ The NRS is a 21-item scale that measures the ‘affective, cognitive and physical relationship individuals’ have with the natural world. For the purpose of the previous pilot study,^25^ the NRS was translated and validated in the Swedish language (results not published).

Rationale: In line with guidance and instructions for RCTs on complex interventions,^30^ nature connectedness is hypothesised to be the underlying mechanism (intermediate outcome) along the theorised impact path towards eudaemonic (psychological) well-being. A significant increase in NRS has previously been demonstrated in childhood and AYA cancer survivors after participation in the WAYA programme compared with the hotel stay.^25^

#### Secondary outcomes (exploratory)

Secondary outcomes explore possible changes in quality of life, physical activity and fitness:

1. Changes in quality of life of AYAs affected by cancer over time, measured by the Minneapolis Manchester Quality of Life instrument (MMQL).^34^ The MMQL is a quality-of-life questionnaire specifically designed for cancer survivors and consists of seven quality of life domains: physical functioning, cognitive functioning, psychological functioning, body image, social functioning, intimate relations and outlook on life. The instrument is translated and validated in the Swedish context.
2. Changes in light and moderate-to-vigorous intensity physical activity will be measured objectively using ActiGraph.^35^
3. Changes in maximal oxygen consumption (VO2max; mL/kg/min) will be estimated by means of the Ekblom Bak cycle ergometer test.^36^
4. Blood pressure and heart rate will be assessed with an electronic monitor in a seated position after a 5 min rest. Two measurements will be performed, and the lowest value will be registered.
5. Body mass index (BMI) will be measured non-fasted and in light clothing to the nearest 0.1 kg by a digital scale. Height will be measured to the nearest 1 mm by a stadiometer. BMI (kg/m^2^) will be calculated.

Considering the large similarity between the Swedish and Norwegian language, the validated Swedish primary outcome scales (PWS, NRS and MMQL) will only be translated to the Norwegian language and back translated for quality control.

#### Safety-related outcomes

An occurrence of (serious) AEs during the interventions is reported and documented in the field diaries. AEs will be coded according to the Medical Dictionary for Regulatory Activities^37^ and analysed using preferred terms and allocation to system organ class. The safety of the programmes will be evaluated by analysing the number, seriousness, intensity and types of AEs that are evaluated to be certain, probable/likely or possibly related to the study programmes. Equal safety between the wilderness programme and hotel stay is defined as no significant differences in the risk of having serious AEs on wilderness versus hotel stay participation.

### Data collection

Table 1 gives an overview of time points for data collection in the proposed study.

**Table 1:** Timeline of measurements in the study

Measurements	Screening	Randomisation	Intervention 1		3 months at home		Intervention 2	1 year
	Contact 1	Contact 2	Day 1 T=0*	Day 8* T=1*	Contact 3	Contact 4	Month 3 T=2*	Year 1 T=3
Primary outcomes								
PWS			X	X			X	X
NRS			X	X			X	X
Secondary outcomes								
MMQL			X	X			X	X
Physical activity			X	X			X	X
VO2max			X				X	
Blood pressure			X				X	
Heart rate			X				X	
Body mass index			X				X	
Adverse effects				X	X	X	X	X
Other data								
Willingness	X							
Preference	X							
Expectations	X							
Adherence				X			X	
Effectiveness		X			X	X		X
Demographic data	X							
Medical history		X						
Medication use		X			X	X		X
Other therapies/ self-care		X			X	X		X
Lifestyle changes		X			X	X		X
Supportive relationships		X			X	X		X
Self-efficacy outdoors		X			X	X		X
Outdoor activities		X			X	X		X
Physical activities		X			X	X		X

*To avoid possible effects of intervention-related expectations and anxiety and return euphoria, all self-reported questionnaires will be filled in by participants 2–3 weeks before the start of the first intervention, 2–3 weeks after intervention and 2–3 weeks after the second intervention. Willingness: participants’ willingness to be randomised, Preference: participants’ intervention preference, expectations: participants’ intervention expectations, Adherence: participants’ intervention adherence, Effectiveness: participants’ expected intervention the effectiveness.

MMQL, Minneapolis Manchester Quality of Life instrument; NRS, Nature Relatedness Scale; PWS, Psychological Well-being Scale; VO2max, maximal oxygen consumption.

The first part of this study has a follow-up time of 1 year. In addition to primary and secondary outcomes, other data will be collected for programme comparison, evaluation, study quality and reporting (see table 1). Lifestyle changes include diet, sleep, stress, physical activity (type, frequency/length), smoking, nicotine use, alcohol, caffeine and outdoor/nature activities (type, frequency/length). Supportive relationships concern the experienced relationships with others affected by cancer (scale 1–5, not at all to very much). Self-efficacy outdoors concern the confidence to spend time in nature/outdoors and their outdoor skills (scale 1–5, not at all to very much).

### Sample size

The current study aims to recruit a total of 150 participants who will be randomised into two groups (n=75 per group). The power calculation is based on a two-group linear mixed-effects model for repeated measures data with attrition^38^ and provides 80% power to detect a medium-sized between-group difference (SMD=0.60). In the sample size estimation, we account for an attrition rate of 34.5% before the start of the intervention as reported in the previous pilot study^25^ and a low attrition rate (10%) during the intervention. The power calculation is based on a model with four repeated measurement points, a first-order autoregressive correlation structure, an initial repeated measures correlation of 0.60 (with decreasing magnitude of the repeated measures correlations with increasing distance between time points), an alpha level of .05, and a group allocation of 1:1.

The effect size estimates for the primary outcomes are based on a meta-analytical estimate^32^ of a standardised between-group difference of 0.69 (Hedges’ g) for interventions to enhance eudaemonic psychological well-being, and findings from a pilot study indicating a significant between-group difference in NRS between the wilderness programme and hotel stay (Cohen’s d=0.63).^25^

### Recruitment

Participants will be recruited among members of the Swedish cancer organisations Ung Cancer and the Childhood Cancer Fund, members of the Norwegian cancer organisation Ung Kreft and through oncology departments of hospitals in Norway and Sweden. In all news items, information and brochures, AYAs are directed to the website of the WAYA-2 study where they can register their interest in possible participation in the study.^39^

### Randomisation and concealment

Participants will be randomised equally (1:1) to the wilderness programme or hotel stay according to a randomisation list as generated by a Random Allocation Software Programme^40^ using a random block size of two to guarantee a balanced allocation. Participants will be stratified according to two age groups (16–30, 31–39 years), binary gender categorisations (male/female) and cancer survivorship (childhood cancer: age 0–14 years vs AYA cancer: age 15–39 years) to achieve equal distribution among the two groups. Per stratification and per country (Norway, Sweden), separate randomisation lists will be generated. AYAs affected by cancer who have registered their interest in the study via the website will receive a screening number based on the date of registration entry. Eligible participants will be randomised and assigned to the wilderness programme or hotel stay based on the fixed order of the screening numbers by a researcher who is not involved in the screening and intake of study participants. Allocation to programme intervention will be concealed by the complexity of the randomisation procedure involving a group of ten or more eligible participants at a time and randomisations over eight different strata during the period of 1 year.

### Blinding

On screening, participants will be informed that two possibly effective programmes will be investigated. To reduce expectation bias, it will not be made known to participants whether the wilderness programme is hypothesised to be better than a hotel stay (single blinded). Statistical analysis will be performed by a statistician who is blind to programme allocation.

### Data management and confidentiality

Study data of each participant will be handled in a case report form (CRF). Self-reported demographic data, medical data (cancer diagnosis, cancer treatment, medical history, etc), and other relevant intervention-related data (preference, expectations, effectiveness, etc) will also be documented in the CRF. Participants are allowed to continue all their medication, therapies and/or dietary and lifestyle measures during the study. Any changes in medication, therapies, dietary and lifestyle measures over time will be documented in the CRF. CRF data will be anonymised for analysis by assigning each participant an unidentifiable three-digit screening number at the time of enrolment and additional three-digit study number on randomisation. The CRF, SPSS dataset and other participant-related documentation will be stored on a secure server at Mid Sweden University or Agder University and can only be accessed with a password by research team members who are authorised. To guarantee data quality, independent checks during data collection and data entry will be performed by another researcher than the one collecting or entering the data in the database. A data monitoring committee will not be established because of the nature of the study (health-promotion intervention with low risk). The collected data will be saved for at least ten years after the end of the study.

### Data analysis

χ^2^ statistics, independent samples t-tests and analysis of variance will be used to calculate group differences in sociodemographic variables and cancer characteristics at baseline. The alpha level will be set to 0.05. The primary analysis will investigate changes in the total scores of the PWS and NRS over time from baseline to 1- year follow-up in the intervention group compared with the control group. Missing data will be imputed by means of multiple imputations or by using full-information maximum likelihood estimation.^41^ The analysis of the primary outcome will be based on a linear mixed-effects model and will be fitted to test intervention effects up to 1 year. Adjustments will be made for age, gender, age at cancer diagnoses and study site. Time, intervention arm, interaction between time and intervention arm, age, gender, age at cancer diagnoses and study site are included as fixed effects, and the participant is included as random effect in the model. Statistical analyses will be on the intention-to-treat principle (all participants who are randomised in the study). As sensitivity analyses, the per-protocol data set will be analysed including only participants who have participated for at least 1 day in the wilderness or hotel stay. Secondary outcomes will also be analysed using linear mixed-effects models.

Categorical data will be presented as frequencies and percentages. For continuous data, the number, mean, SD, median, IQR, minimum and maximum will be calculated. A two-sided p<0.05 will be considered statistically significant.

Safety analyses will be performed on the safety analysis set and include all individuals who had participated in one of the programme interventions for at least 1 day. RR with 95% CI will be calculated to indicate the risk of having an AE adjusted to days of exposure to programme interventions. Pearson’s χ^2^ test will be used to calculate differences in AEs severity between the two study arms. A detailed description of the planned statistical analyses will be provided in the statistical analysis plan, which will be finalised prior to database closure and data analysis. All statistical analyses will be performed by using Stata V.18.0.

### Protocol amendments

Possible changes to this protocol, and reasons for these changes, will be documented in the trial register (www.isrctn.com), and if needed submitted to the Swedish and Norwegian Ethical Review Authority for approval. The registered study protocol (ISRCTN93856392) includes all items on the minimum standard checklist of items of the WHO.^42^

### Patient and public involvement

User involvement and collaboration with the wider community were established during our previous WAYA-1 study and will be continued in this study. Three AYAs affected by cancer are on the advisory board of the WAYA-2 study. For all wilderness and hotel stay interventions, at least one member of the facilitating team will be an AYA affected by cancer. The WAYA-2 study design and recruitment strategies have been developed in close collaboration with AYAs affected by cancer, the Swedish cancer organisations for childhood and young cancer, researchers from four different countries (Netherlands, Norway, Sweden, USA) and healthcare professionals from the region Västernorrland in Sweden. The content of the WAYA-2 programme and hotel stay has been developed in close collaboration with the Swedish survival guilt, other outdoor instructors/nature guides and the Swedish cancer organisation and optimised through feedback from previous participants in the WAYA-1 study. All parties as mentioned above will be involved in interpretation and dissemination of study results.

### Ethics and dissemination

Participants are informed both orally and in writing that participation in the WAYA-2 study is voluntary. It is made clear that participation in all study-related activities is voluntary, that they can decline participation in any activity without explanation, and that they can withdraw at any time from the programme without stating a reason. Participants are further informed about the purpose of the study, that data will be handled and later published and presented in a confidential way. Informed consent will be obtained from all participants in writing prior to the start of the study and sent to the researcher responsible for screening and intake (see online supplemental material 1 for an example of the participant consent form). In Norway and Sweden, participants from age 16 can provide individual informed consent without permission/signature from parents/guardians. Participation in the WAYA programme is without costs for participants.

10.1136/bmjopen-2024-087626.supp1 Supplementary data

The WAYA-2 study will be performed in line with the instructions and precautions described in the study safety plan.^24^ A physician with emergency medicine expertise will be available by telephone in case of emergency. Adequate insurance for all participants and facilitators in the study will be provided by Mid Sweden University or Agder University. The study protocol was approved by the Swedish Ethical Review Authority on 27 September 2023 (reference: 2023-05247-01). The study application to the Norwegian Ethical Review Authority is pending (reference: 694114). Information letters and consent forms will be accessible on request.

We aim for a wide communication and dissemination of research results through close collaboration with national and international research partners and the wider communities in the respective countries. Results of the study will be presented to the participants. It aims to publish at least three scientific (peer-reviewed) articles and to present the results at (scientific) national and international conferences. Popular articles with study results will be disseminated in the form of interviews, books and chronicles. News items will also be spread via social media (Facebook, Twitter), websites and newsletters of collaborating partners and organisations and of the funding bodies.

### Timeline of the study

**Figure 1:**
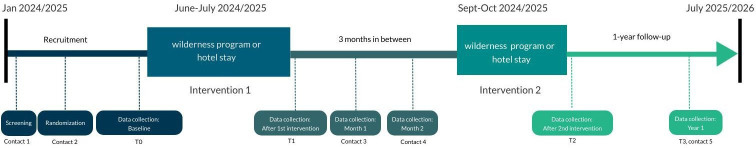
Time line of the WAYA-2 study. WAYA-2, Wilderness Programme for Adolescents and Young Adults.

The timeline of the study is depicted in figure 1. The first participants were enrolled in the study on 19 February 2024. The first interventions in Sweden will be carried out during the summer of 2024. The interventions in Norway will take place in the summer of 2025. Since participants are followed for 1 year after the start of the intervention, data collection will end in July 2026. Data analyses are planned to start in December 2026 and reporting is planned to end in December 2027. It is the aim of this study that participants will be followed up for 10 years after the study starts (2, 5 and 10 years follow-up) to investigate possible long-term changes in psychological well-being, health-related quality of life and physical activity. Ethical approval and finance for the long-term follow-up of the WAYA-2 study will be sought in 2025.

## Discussion

AYAs affected by cancer are an understudied and vulnerable group and effective interventions are needed to ensure that they are able to cope with late effects of cancer, its treatment and achieve good quality of life. To the best of our knowledge, worldwide this study will be the first full-scale RCT that investigates the effectiveness of a WAYAs affected by cancer, and the first wilderness programme in Scandinavia that is developed and adapted to their needs. The WAYA programme in this study is specifically designed to offer a supervised wilderness programme to AYAs with a wide range of disabilities.^24^

This study aims to demonstrate the evidence base for a wilderness programme intending to impact the psychological well-being of AYAs affected by cancer. The expected short-term impact is that the wilderness programme may support their self-efficacy, self-confidence and provide them with the competence to develop and maintain their own outdoor practice. The long-term impact of the wilderness programme may decrease depression, anxiety, and stress, and increase quality of life. In the development of the previous pilot RCT design, considerable effort was invested to define an appropriate control group activity.^21^ A hotel stay was chosen to control for attention from facilitators, group support and being away from their home environment. Although it might be expected that relaxation and connecting with other AYAs offer positive experiences and time to recharge, it is expected that these may have less impact on their health and well-being in the long term.^43 44^ Based on previous experiences with the feasibility of a planned RCT in Norway,^45^ it was decided not to include a ‘care as usual’ or wait-list control arm in the WAYA-2 study.

This study is expected to fill a gap in the literature by providing valuable insights and needed evidence on the role of nature in the support of the health and well-being of such a vulnerable and understudied population. This will inform AYAs affected by cancer, their families, outdoor instructors, clinicians and other researchers.

One of the expected challenges of this study is to recruit the needed number of participants in the study within the set time frame. We previously published that the eligibility (87.3%) and enrolment rate (95.2%) of participants in the WAYA-1 study were high.^25^ To randomise 150 eligible individuals into the WAYA-2 study, we, therefore, need to recruit at least 180 AYAs affected by cancer to register their interest to participate in the WAYA-2 study. In our previous pilot study, we only recruited among members of Swedish cancer organisations Ung Cancer and the Childhood Cancer Fund, and the recruitment rate was low (3.6%).^25^ However, the pilot study was performed during the COVID-19 pandemic where recommendations with respect to travel restrictions were in place. It is, therefore, expected a higher recruitment rate in the WAYA-2 study. Furthermore, we will now also recruit participants in Norway and additional recruitment strategies will be developed with the support of healthcare professionals and brochures in waiting rooms of oncology departments of hospitals in Norway and Sweden.

Another potential challenge of the WAYA-2 study is the inclusion of a heterogeneous study population (all cancer diagnosis and all cancer treatment stages) with a wide age range (16–39 years). Programme activities and study outcomes may be affected by participants being at very different stages of their cancer trajectory and/or lives. The inclusion of participants with a large span was investigated in detail in the previous pilot study and results have been published.^24^ It appeared that the wide age range and different stages in the cancer process were perceived to be an advantage (rather than a disadvantage) since it positively influenced group bonding and learning process, according to facilitators and participants.

This study involves a unique cross-sectional international (Norway, Sweden, USA) and multidisciplinary collaboration between academic researchers, clinicians, outdoor instructors, public health professionals, patient organisations and non-profit organisations in the outdoor sector. Members of the research team have expertise in clinical trials, epidemiology, nature-based therapies, nursing, oncology, psycho-oncology, public health, social sciences, sport sciences, wilderness therapy and have previously published on the topic.^20 21 24–26 45–49^ It is expected that such a collaboration is needed to tackle the complexity of this multicomponent intervention for AYAs affected by cancer.

### Study status

First participants were enrolled in the study on 19 February 2024. The estimated end of the first part of the study (1-year follow-up) is planned on 31 December 2027.

## Supplementary Material

Reviewer comments

Author's manuscript
