# Supplementary material for: Protocol of a randomised controlled multicentre trial investigating the effectiveness and safety of a wilderness programme on the mental and physical well-being of adolescents and young adults affected by cancer: the WAYA-2 study
Source: BMJ Open. 2024 May 21;14(5):e087626. doi: 10.1136/bmjopen-2024-087626 (PMC11110565; doi:10.1136/bmjopen-2024-087626)
Supplement: Supplementary data [file bmjopen-2024-087626supp001.pdf]

En studie som undersöker säkerhet samt vilka effekter naturvistelse och vila/rekreation har på mentalt och psykiskt välmående bland unga canceröverlevare

Mittuniversitetet
MID SWEDEN UNIVERSITY
Institutionen för Hälsovetenskaper

Information till deltagare

En studie som undersöker säkerhet samt vilka effekter naturvistelse och vila/rekreation har på mentalt och psykiskt välmående bland unga canceröverlevare

Du har visat intresse för att om möjligt delta i den här studien.

Innan Du slutligen bestämmer Dig, är det viktigt att Du får veta mer om vad studien innebär. Av den anledningen är det viktigt att Du läser detta brev noggrant. Prata med din familj eller vänner, och om Du har frågor efter att ha läst informationsmaterialet så är Du välkommen att kontakta oss (ansvariga forskare, kontaktuppgifter i slutet av informationsbrevet).

De senaste åren har det bland människor i allmänhet samt inom forskning mer och mer uppmärksammats att vistelse i naturen kan bidra till hälsa och välmående.
Naturbaserade program ses som lovande vägar för att stötta ungdomar hälsa och välmående i allmänhet men det finns mycket litet forskning kring hur det fungerar/upplevs hos unga canceröverlevare och man vet inte säkert om de resutat som ses beror på om deltagare har en möjlighet till att komma bort från sin hemmiljö och slappna av, eller om det är just naturvistelse som ger känsla av välmående.

Vad är syftet med studien?

Studien är en så kallad randomiserad kontrollerad studie, där deltagarna lottas till att antingen delta i ett naturbaserat friluftsprogram eller ett semesterprogram. I studien kommer vi att undersöka om det finns skillnader mellan grupperna avseende förändringar i hälsa och välmående. Det kommer även att undersökas om båda programmen är lika säkra att genomföra.

Vem kan vara med?

Är du mellan 16-39 år gammal och är/har varit drabbad av cancer någon gång i ditt liv så kan du vara med.

Andra krav:

- Att du klara av att gå minst 2 km utan paus (kryckor som gånghjälpmedel går bra)
- Att du pratar och förstår antingen svenska, norska, danska eller engelska
- Att du accepterar att du kommer att bli lottad till deltagande i den ena eller andra gruppen

Du kan vara med även om du har andra fysiska eller psykiska funktionsnedsättningar, syn, hörsel etc, eller om du ej kan bära en ryggsäck – då ordnar vi med stöd/ledsagare.

Av säkerhetsskäl kan du ej vara med om du har ett sjukdomstillstånd eller nyligen genomgått cytostatikabehandling som innebär extra hög blödningsrisk eller infektionskänslighet – om det finns frågetecken kring det så måste vi rådgöra med din behandlande läkare för att avgöra om deltagande är säkert för just dig.

Hur går det till och vad innebär deltagandet

Totalt i studien kommer det att ingå 150 unga canceröverlevare 16-39 år gamla som rekryterats med hjälp av föreningarna Ung Cancer, Maxa Livet (Sverige, Ung Kreft (Norge), Ung Kræft (Danmark) bland föreningen Ung Cancers medlemmar. Hälften av dessa (75 personer) kommer att lottas till att delta i programmet som innehåller naturvistelse, och hälften (75 personer) till semestervistelse (en

avslappnande semestervecka på hotell med spa). Studien genomförs parallellt i Sverige och Norge, vilket innebär att deltagarna i Sverige (cirka 75) i huvudsak kommer att vara från Sverige.

Alla deltagare kommer att genomgå (enklare) fysiska tester avseende kondition, samt få besvara frågeformulär kopplat till grundläggande medicinsk bakgrund, hälsa och välmående både före, direkt efter, tre månader efter samt ett år efter genomgånget program.

Vidare kommer deltagarna även att intervjuas avseende deras upplevelser i samband med deltagande – detta sker i samband med tremånadersuppföljning samt ett år efter programmet startade.

Programmen

Det naturbaserade friluftsprogrammet

Deltagarna som blir lottade till friluftsprogrammet kommer i grupper om tio personer att få delta i en åtta dagars vandring med övernattning i tält (Höga Kusten, Ångermanland). Under veckan genomförs dagligen olika former av gruppövningar. Projektet tillhandahåller all nödvändig friluftsutrustning och mat under de åtta dagarna.

Semesterprogrammet

Deltagarna i semesterprogrammet kommer i grupper om tio personer få tillbringa en avslappnande semestervecka på ett hotell där det bland annat finns möjlighet till bad och spa faciliteter.

Tremånader uppföljning av båda grupperna

Båda grupperna följs under tre månader efter första programveckan och kommer att få besvara samma webbaserade frågeformulär som före programmet. Alla kommer efter tre månader även få komma ”tillbaks” för en uppföljningsträff, med ytterligare en kort vandring med övernattning i naturen för friluftsgruppen, samt avslappnande hotell/spaweekend för semestergruppen.

Programteam

Vid båda programmen kommer minst tre handledare/instruktörer att vara närvarande för att stötta och guida deltagarna. Bland dessa finns stor frilufts- och sjukvårdskompetens (Sjuksköterskor/psykolog/läkare) och det finns även expertkompetens inom cancervård knutet till teamet (Läkare).

Kostnadsfritt deltagande

Allt deltagande i studien är kostnadsfritt och projektet kommer att stå för resekostnader, mat och utrustning under deltagandet.

Projektet tecknar även en specifik olycksfallsförsäkring via Kammarkollogiet för alla deltagare under den tid de reser till/från event, samt under event.

Ersättning ges inte för eventuell förlorad arbetsinkomst.

Hur mycket tid tar deltagande för dig och vilka risker innebär deltagande

Om Du väljer att delta förväntar vi oss att Du under ett års lägger ner en del tid för att fylla i alla enkäter, samt även för resor till och från event (8+4 dagar).

Enkäterna är webbaserade och besvaras före programdeltagande, två veckor efter, två veckor efter tremånadersuppföljning, samt ett år efter start i studien.

Att fylla i de webbaserade enkäterna tar cirka 30 minuter per tillfälle och vid två tillfällen kommer du även att få genomföra en enklare konditionstest som tar cirka 30 minuter. Om vi upptäcker avvikande resultat i exempelvis blodtryck, så gör dig uppmärksam på det så att du kan kontakta din primärvårdsläkare för uppföljning.

Så vitt vi känner till så innebär deltagandet i studien inga extra risker för Dig utom för vad som normalt kan förväntas när personer vistas ute i skog och mark, möjligen liten risk för: träningsvärk, skavsår, vrickning, solexponering mm. Dock kommer teamet bistå i den mån det går till att förebygga att det uppstår.

Om det till följd av din cancersjukdom (eller behandling) finns extra risker för just dig är det viktigt att vi känner till dem och eventuellt måste rådgöra med din behandlande läkare.

Dina rättigheter

Deltagandet är **frivilligt** och Du kan själv välja att avstå deltagande när som helst, utan att ange skäl.

Dina svar och dina resultat kommer att behandlas så att inte obehöriga kan ta del av dem. Vid rapportering och publicering av forskningsresultat, (som kommer att ske i nationella och internationella vetenskapliga tidskrifter och populärvetenskapliga texter), kan inga enskilda individer bli identifierade i redovisningen.

Efter studiens avslut skickas rapporter och artiklar ut till de deltagare som önskar få dem.

Forskningshuvudman för projektet är Mittuniversitetet. Med forskningshuvudman menas den organisation som är ansvarig för projektet. Ansökan är godkänd av Etikprövningsmyndigheten, diarienummer för prövningen hos Etikprövningsmyndigheten är 2023-05247-01.
Dina svar och dina resultat kommer att behandlas så att inte obehöriga kan ta del av dem.
Ansvarig för dina personuppgifter är Mittuniversitetets dataskyddsombud. Enligt EU:s dataskyddsförordning har du rätt att kostnadsfritt få ta del av de uppgifter om dig som hanteras i projektet, och vid behov få eventuella fel rättade. Du kan också begära att uppgifter om dig raderas samt att behandlingen av dina personuppgifter begränsas. Rätten till radering och till begränsning av behandling av personuppgifter gäller dock inte när uppgifterna är nödvändiga för den aktuella forskningen. Om du vill ta del av uppgifterna ska du kontakta huvudansvarig forskare (se kontaktuppgifter nedan). Dataskyddsombud nås på 060-142 80 00 eller via e-post: dataskyddsombud@miun.se. Om du är missnöjd med hur dina personuppgifter behandlas har du rätt att ge in klagomål till Integritetsskyddsmyndigheten, som är tillsynsmyndighet.

Om du har frågor eller funderingar som du vill diskutera så är Du välkommen att ringa någon av studieansvariga

Studien är ett internationellt samarbetsprojekt med deltagande forskare från Mittuniversitetet i Sverige, Universitetet i Tromsø, Universitetet i Agder och Sørlandets Sjukhus, Kristiansand i Norge, samt från University of California San i USA. Andra samverkanspartners i projektet är: Föreningarna Ung Cancer, Maxa Livet, Ung Kreft (Norge), Ung Kræft (Danmark), Svenska Överlevnadssällskapet och Region Västernorrland.

Mats Jong

Docent i Omvårdnad, Leg Sjuksköterska
Institutionen för Hälsovetenskaper/Folkhälsa
Mittuniversitetet, Sundsvall
Tel: 010/1428966, 072/581 896 6
email: mats.jong@miun.se

Miek Jong

Docent i Hälsovetenskap
Institutionen för Hälsovetenskaper/Folkhälsa
Mittuniversitetet, Sundsvall
Tel: 010/1428967
email: miek.jong@miun.se

Mittuniversitetet
MID SWEDEN UNIVERSITY
Institutionen för Hälsovetenskap

Samtyckesformulär avseende deltagande i studie som undersöker säkerhet samt vilka effekter naturvistelse har på mentalt och psykiskt välmående bland unga canceröverlevare

Jag är tillfrågad om att delta i denna vetenskapliga studie

Namn:____

- Jag har läst informationsbrevet till deltagare. Jag hade möjlighet att ställa ytterligare frågor och dessa besvarades tillfredsställande, jag hade även tillräckligt med tid för att avgöra om jag ville delta eller inte, och får även behålla den skriftliga informationen.
- Jag förstår att deltagande är helt frivilligt samt att jag kan besluta mig om att avsluta mitt deltagande när som helst utan studiens gång utan att ange orsak.
- Jag medger att det insamlade data får användas för det syfte som beskrivets i informationsbrevet.
- Jag är medveten om att det insamlade materialet kommer att sparas i minst 13 år på Mittuniversitetet i Sundsvall innan det får förstöras.
- Jag samtycker till att ansvariga forskare i studien kan kontakta mig efter studiens avslutande för att höra efter hur det går för mig.

- Jag samtycker till att ansvariga forskare i studien kan kontakta mig för att tillfråga mig om deltagande i en uppföljningsstudie i framtiden.
  ¨Ja ¨Nej

- Om det finns tveksamheter avseende om min hälsosituation medger studiedeltagande samtycker jag till att informera min behandlade läkare om mitt deltagande. I detta avseende samtycker jag även till att studieansvarig forskare/forskningskoordinator får etablera kontakt med denna för att säkerställa att det inte föreligger direkta medicinska hinder för mig att delta i studien.
- Jag önskar delta i denna studie.

Studiedeltagarens namn:_________________________Personnummer:______________-____

Underskrift:__________________________________ Datum:________________________

Jag bekräftar att jag har tydligt och uttömmande informerat ovan nämnda person om detta forskningsprojekt. Om det under studiens gång framkommer uppgifter som kan påverka personens samtycke till deltagande, så kommer jag att informera henne så fort som möjligt

Forskarens namn:______________________________

Underskrift:____________________________________ Datum:_______________________

Jong MC, *et al. BMJ Open* 2024; 14:e087626. doi: 10.1136/bmjopen-2024-087626
