# Supplementary material for: Protocol of a randomised controlled multicentre trial investigating the effectiveness and safety of a wilderness programme on the mental and physical well-being of adolescents and young adults affected by cancer: the WAYA-2 study
Source: BMJ Open. 2024 May 21;14(5):e087626. doi: 10.1136/bmjopen-2024-087626 (PMC11110565; doi:10.1136/bmjopen-2024-087626)
Supplement: Author's manuscript [file bmjopen-2024-087626.draft_revisions.pdf]

BMJ Open is committed to open peer review. As part of this commitment we make the peer review history of every article we publish publicly available.

When an article is published we post the peer reviewers' comments and the authors' responses online. We also post the versions of the paper that were used during peer review. These are the versions that the peer review comments apply to.

The versions of the paper that follow are the versions that were submitted during the peer review process. They are not the versions of record or the final published versions. They should not be cited or distributed as the published version of this manuscript.

BMJ Open is an open access journal and the full, final, typeset and author-corrected version of record of the manuscript is available on our site with no access controls, subscription charges or pay-per-view fees (http://bmjopen.bmj.com).

If you have any questions on BMJ Open's open peer review process please email info.bmjopen@bmj.com

BMJ Open

Protocol of a randomized controlled multi-centre trial investigating the effectiveness and safety of a wilderness program on the mental and physical well-being of adolescents and young adults affected by cancer: the WAYA-2 study.

Journal:	*BMJ Open*
Manuscript ID	bmjopen-2024-087626
Article Type:	Protocol
Date Submitted by the Author:	15-Apr-2024

Complete List of Authors:	Jong, Miek; Mid Sweden University, Department of Health Sciences; UiT The Arctic University of Norway Faculty of Health Sciences, Department of Community Medicine, National Research Center in Complementary and Alternative Medicine (NAFKAM)<br>Fernee, Carina Ribe; Sorlandet Hospital Kristiansand, Department of Child and Adolescent Mental Health; University of Agder, Department of Sport Science and Physical Education<br>Stenling, Andreas; University of Agder, Department of Sport Science and Physical Education; Umeå University, Department of Psychology<br>Lown, E. Anne; University of California San Francisco, Department of Social & Behavioral Sciences<br>Berntsen, Sveinung; University of Agder, Department of Sport Science and Physical Education<br>Victorson , David; Northwestern University - Chicago Campus, Department of Medical Social Sciences, Feinberg School of Medicine<br>Jong, Mats; Mid Sweden University, Health Sciences

Keywords:	Safety, Quality of Life, ONCOLOGY, Psychosocial Intervention, Self-Management

SCHOLARONE™
Manuscripts

Protocol of a randomized controlled multi-centre trial investigating the effectiveness and safety of a wilderness program on the mental and physical well-being of adolescents and young adults affected by cancer: the WAYA-2 study.

Miek C. Jong[1,2], Carina Ribe Fernee[3,4], Sveinung Berntsen[4], Andreas Stenling[4,5], David E Victorson[6], E. Anne Lown[7], Mats Jong[1]

[1]Mid Sweden University, Department of Health Sciences, Sundsvall, Sweden.

[2]The Arctic University of Norway, National Research Center in Complementary and Alternative Medicine (NAFKAM), Department of Community Medicine, Faculty of Health Sciences, UiT, Tromsø, Norway.

[3]Department of Child and Adolescent Mental Health, Sorlandet Hospital, Kristiansand, Norway.

[4]Department of Sport Science and Physical Education, University of Agder, Kristiansand, Norway.

[5]Department of Psychology, Umeå University, Umeå, Sweden.

[6]Department of Medical Social Sciences, Feinberg School of Medicine, Northwestern University, Chicago, IL, USA.

[7]Department of Social & Behavioral Sciences, University of California San Francisco, San Francisco, USA.

Correspondence to:

Miek C. Jong, The Arctic University of Norway, National Research Center in Complementary and Alternative Medicine (NAFKAM), Department of Community Medicine, Faculty of Health Sciences, UiT, Hansine Hansens veg 18, 9019 Tromsø, Norway.

T: +46 10 142 89 67, email address: miek.jong@uit.no

For peer review only - http://bmjopen.bmj.com/site/about/guidelines.xhtml

Keywords: Oncology, psychosocial intervention, safety, self-management, quality of life

Word count: 5213

Date/version: 05-04-2024, version 6

ABSTRACT (max 300 words): 297

Introduction

Adolescent and young adults (AYAs) affected by cancer are an understudied group. Effective interventions are needed to support coping with late effects of cancer, its treatment, and to promote quality of life. Nature-based interventions may be promising in support of the self-management and health of AYAs affected by cancer. However, randomized controlled studies (RCTs) on the effectiveness of such interventions are lacking. We performed a first pilot RCT (n=42) that showed that it is feasible and safe to conduct such a study. Here we propose a full scale RCT to investigate the effectiveness and safety of a wilderness program on the mental and physical health of AYAs affected by cancer.

Methods and analysis

Participants are 150 AYAs affected by cancer, aged 16-39 years, who will be randomized to a wilderness (n=75) or a hotel stay (n=75). The wilderness program is an eight-day intervention including a six-day wilderness expedition. This is followed three months later by a four-day intervention including a two-day basecamp. Activities include hiking, backpacking, kayaking, rock climbing, mindfulness, and bush-crafting. The comparison group is an eight-day hotel stay followed by a four-day hotel stay (interventions include two travel days) at the same hotel after three months. Primary outcomes are psychological well-being and nature connectedness up to one year after study start. Secondary outcomes are quality of life, physical activity, and safety parameters.

For peer review only - http://bmjopen.bmj.com/site/about/guidelines.xhtml

Ethics and dissemination

The Swedish Ethical Review Authority approved the study protocol on 27 September 2023 (reference: 2023-05247-01). The recruitment started on 19 February 2024 and the first part is planned to end on 31 December 2027. Study results will be disseminated by means of scientific publications, presentations at conferences, popular articles, interviews, chronicles, and books. News items will be spread via social media, websites, and newsletters.

Trial registration number ISRCTN93856392 (www.isrctn.com)

Strengths and limitations of this study

- The WAYA program has previously been piloted and was found to be safe, developmentally appropriate, and of interest to the population under investigation [1, 2].
- Worldwide, this will be the first randomized-controlled trial investigating the effectiveness and safety of a wilderness program for adolescent and young adults affected by cancer.
- This study aims to investigate long-term health effects of a wilderness program, up to ten years after the intervention.
- This study will include a heterogeneous cancer study population regarding age (16-39 years), age at diagnosis (0-39 years), diagnosis (all), and stage of treatment (active treatment and survivorship).
- A potential third arm, i.e. comparison to no program participation or a wait-list control, will not be included in this randomized-controlled trial.

For peer review only

For peer review only - http://bmjopen.bmj.com/site/about/guidelines.xhtml

"The smaller we come to feel ourselves compared to the mountain, the nearer we come to participating in its greatness. I do not know why this is so"

Arne Naess

INTRODUCTION

Adolescent and young adults (AYAs) affected by cancer, either by a childhood or AYA cancer diagnosis, are a growing but understudied population [3]. Due to cancer or its treatment, many AYAs experience at least one late effect such as psychosocial problems, concentration/memory difficulties and fatigue that negatively impact their quality of life [4]. Furthermore, they suffer disproportionately from chronic diseases and have excess risk for early mortality compared to non-cancer populations [5]. It appears that AYAs affected by cancer receive little follow-up care that appropriately address these late effects [4]. Consequently, different self-management strategies have evolved by which they are trying to cope with late effects. These include pushing themselves physically and mentally to master everyday activities, balancing social activities and rest, and recuperating and regaining energy [6]. Other coping strategies reported are seeking distraction, support from family and friends, yoga or meditation, or changes in lifestyle such as increased physical activity and healthy diets [7]. Although AYAs affected by cancer acknowledge the importance of these self-management strategies in dealing with late effects of cancer or the cancer treatment itself, it remains an ongoing challenge [7]. Therefore, it is a high priority to find effective interventions that support the self-efficacy of AYA impacted by cancer, thereby minimizing the negative impact of cancer-related late effects and improving their quality of life [7].

Physical activity-based interventions have been shown to increase physical activity and reduce fatigue in childhood cancer survivors [8-10]. However, results must be interpreted with caution due to the small sample sizes and large heterogeneity among the reviewed studies [9]. Physical activity-based interventions have also been reported to increase the

For peer review only - http://bmjopen.bmj.com/site/about/guidelines.xhtml

quality of life of AYA cancer survivors, however, with marginal significance [11]. It appears to be difficult for AYAs affected by cancer to return to or maintain adequate levels of physical activity, and they seem to make little use of existing supporting physical activity services within the community [12]. A number of barriers exist that make it more difficult to engage in physical activity including self-reported fears about health, low baseline activity levels and/or fitness, lack of motivation, cancer-related pain or fatigue, age at diagnosis, anxiety about health, and lack of advice from a physician [13, 14].

Another promising intervention in support of the self-management of cancer survivors are nature-based interventions. Those affected by cancer have reported that engaging with nature is important for their health and well-being [15-17]. Research from different fields indicate that nature-based interventions may decrease anxiety, depression, stress, tension, fatigue, pain, and improve sleep in those affected by cancer [18]. Nature may provide a safe space and motivational context for cancer patients that supports relaxation, recovery, physical activity and may ignite reflection, inspiration, personal growth, and supportive relationships [16, 19, 20]. Furthermore, it has been demonstrated that spending time in nature leads to more frequent or longer stays in nature [21].

To gain insight into possible beneficial effects of nature-based interventions specifically for AYAs affected by cancer, we first performed a scoping review on the topic [22]. Nature-based interventions such as adventure and wilderness programs were found to increase self-efficacy, self-esteem, self-confidence, physical activity, and social support among AYAs affected by cancer. However, it was found that randomized controlled trials (RCTs) on effectiveness of nature-based interventions were lacking. Other gaps identified were the lack of studies on the long-term effects (> 3 months) of nature-based interventions, and lack of data on the safety of these interventions [22]. Therefore, in 2021-2022, we performed a pilot study to investigate whether it would be feasible to perform a RCT design on a nature-based

For peer review only - http://bmjopen.bmj.com/site/about/guidelines.xhtml

intervention [23]. For this pilot study we developed a Wilderness program for AYAs (WAYA program) affected by cancer. The WAYA program is grounded in Næss's ecosophy [24] and the Positive Health Model [25]. A detailed description and schematic outline of the WAYA program theory and content has previously been published [2]. We demonstrated that it was feasible and safe to conduct a pilot RCT [1]. None of the 42 AYAs that participated in the interventions dropped-out from the study, and adherence to program interventions and data completeness after one-year follow-up was high. The WAYA program was found to be just as safe for AYAs affected by cancer as a hotel stay. The relative frequency of reported adverse effects (AE) was similar in both study arms (Relative Risk: 1.0, 95% Confidence Interval 0.8-1.3), and all AEs were non-serious, mild, or moderate of severity, and resolved during the study period [1]. Furthermore, the WAYA program appeared to be acceptable, developmentally appropriate and of interest to AYA affected by cancer [1, 2]. Based on these observations we recommended for a future larger RCT to: 1) Apply additional recruitment strategies for participants, 2) To stratify for the inclusion of cancer survivorship (childhood cancer versus AYA cancer), 3) To optimize the content of the WAYA program and 4) To select health-related outcomes based on the lived experiences of participants in the WAYA program [26].

The aim of the present study is to perform a full-scale multi-centre RCT (WAYA-2 study) that investigates the effectiveness and safety of a wilderness program on the mental and physical well-being of AYAs affected by cancer. The WAYA program and WAYA-2 study protocol have been optimized according to the findings and recommendations from the pilot study as described above.

For peer review only - http://bmjopen.bmj.com/site/about/guidelines.xhtml

METHODS AND ANALYSIS

Study guidance and design

The WAYA-2 study protocol is developed in line with the Standard Protocol Items: Recommendations for Interventional Trials (SPIRIT) 2013 guidance for protocols of clinical trials and the SPIRIT 33-item checklist [27]. The WAYA-2 study will be conducted in accordance with the guidelines of the Declaration of Helsinki, and results will be reported according to the Consolidated Standards of Reporting Trials (CONSORT), 25-item checklist and flow diagram [28]. This is a multi-centre RCT in Norway and Sweden with a two-armed parallel design using individual randomization. A quantitative RCT design will allow the testing of active aspects of a complex wilderness intervention including physical activity, physical challenges, experiential activities, being in nature and other reflective practices. A hotel stay is chosen as a comparison to control for other possible effective factors such as attention, group support and getting out of their regular environment.

Research questions

The following research questions will be addressed:

Does a wilderness program, including a wilderness expedition and basecamp stay:

1. Increase psychological well-being of AYAs affected by cancer compared to a control activity?

2. Increase nature connectedness of AYAs affected by cancer compared to a control activity?

3. Improve health-related quality of life of AYAs affected by cancer compared to a control activity?

4. Increase physical activity level of AYAs affected by cancer compared to a control activity?

5. Is a wilderness program equally safe for AYAs affected by cancer as a hotel stay (control activity)?

For peer review only - http://bmjopen.bmj.com/site/about/guidelines.xhtml

Participants and inclusion criteria

Participants are adolescents and young adults aged 16-39 years, that have been diagnosed with any type of cancer during childhood, adolescence, or young adulthood. Another inclusion criterium is the ability to walk 2 km a day (walking aids or assistance from another person is permitted). Participants with various disabilities such as mobility, vision and hearing impairments, balance problems and special treatment or dietary needs can be included. Prior experience with outdoor activities is not required for study participation.

Exclusion criteria are:

- Cancer treatment that imposes unwanted risks for AYAs upon program participation, as evaluated by their treating physician/oncologist.
- A medical condition of AYAs that prevents safe travel to, or participation in the program, as evaluated by their treating physician/oncologist.
- Not willing to be randomly assigned to program interventions.
- Previous participation in the WAYA-1 study.

Following the recommendations of AYA cancer experts in our advisory board, we will deliberately refer to participants in the WAYA-2 study as AYAs affected by cancer, rather than childhood and AYA cancer survivors.

Intervention group: Wilderness program

The development of the Wilderness program for Adolescent and Young Adults (WAYA program) affected by cancer has previously been published [2]. The WAYA program aims to increase physical activity, self-confidence, self-efficacy and self-care, to support personal growth and building meaningful relationships, and to provide an enjoyable and safe stay in nature. Nature in the WAYA program has both a contextual and an active role in support of the health and well-being of AYAs affected by cancer, wherein their health is regarded to be

For peer review only

intrinsically interwoven in a bi-directional fashion with the health and well-being of nature [29]. Participants are given the opportunity to experience a diversity of natural landscapes, and specific nature characteristics are actively incorporated in program activities. Activities in the program include hiking, sea-kayaking, backpacking, rock climbing, camping. The program also includes (nature) reflective practices such as mindfulness, meditation, and forest bathing [2]. Outdoor skills taught include mapping/compass/orienting, trail cooking, safety training, equipment planning, foraging, fishing, bush-craft skills and leave no trace. The wilderness program consists of three intervention parts: 1) An eight-day intervention that includes two days for traveling and physical testing and a six-day wilderness expedition; 2) A three-month in-between period where participants are contacted once every month online to coach them to engage in their own outdoor activities; 3) A four-day intervention including a two-day base camp program. Both the expedition and basecamp program will take place in nature settings around the High Coast of Sweden or in Southern Norway. The group size will be 10-12 participants, and the facilitator-participant ratio will be 3:10. The six-day wilderness expedition will be logistically supported by a team of 5-7 volunteers.

Control group: Hotel stay

The hotel stay will be for eight-days at a Spa Hotel in Sweden or Norway, including two days for traveling and physical testing and a six-day hotel stay, followed by a second four-day stay at the same hotel (including two travel days) three months later. Similar to the wilderness intervention, during the three-month in-between period, participants will be contacted once every month to ask how they are doing. The content of the hotel stay includes the following activities: Spa facilities, museum visits, watching tv/movies, playing games/gaming, shopping, and fine dining. Participants will engage in organized group activities, but this does not include any nature-based activities. The group size is 10-12 participants, and the facilitator-participant ratio will be 3:10.

For peer review only - http://bmjopen.bmj.com/site/about/guidelines.xhtml

Program facilitators

In Sweden and Norway, we will work with professional teams of outdoor instructors and facilitators that have previous experience in wilderness programs with young cancer survivors or other vulnerable youth populations. Facilitators of the WAYA program have competence in one or more of the following areas: outdoor life, survival, nature/wilderness guiding, kayaking, climbing, nursing, first aid, (youth/group) counselling/supervising, mindfulness, mind-body techniques, and data collection for research purposes [2]. At least two facilitators of the WAYA program are licensed health professionals (nurses, psychologists) and at least one facilitator of the WAYA program and hotel stay is an AYA affected by cancer. For program intervention fidelity, referring to the degree to which the WAYA program is performed in a similar manner in Sweden and Norway, two members of the Norwegian outdoor team will join the Swedish outdoor team for the first wilderness intervention in Sweden, and vice versa. For supervision of participants in the hotel stay, facilitators will have competence in guiding/supervising of groups, first aid, and research methodology for data collection and safety monitoring.

Outcomes

The selection of outcomes is driven by the aims of the WAYA program and recently published qualitative study results of the WAYA program [26]. The aims of the WAYA program and underlying process to support self-realization via nature connectedness is hypothesized to contribute to a chain of actions that impacts the six domains of Positive Health [25]. The WAYA program is a complex intervention and may potentially impact on multiple factors. Therefore, in line with guidance and instructions for RCTs on complex interventions, more than one primary outcome is chosen in relation to the research questions [30].

For peer review only - http://bmjopen.bmj.com/site/about/guidelines.xhtml

Primary outcomes (confirmatory)

1. Changes in psychological well-being of AYAs affected by cancer over time, measured by the Psychological Well-being Scale (PWS) [31]. This scale consists of six domains: Self-acceptance, positive relations with others, autonomy, environmental mastery, purpose in life, and personal growth. Together, these dimensions can contribute to the assessment of a person's level of positive functioning and well-being. This instrument is translated and validated in the Swedish context.

Rationale: Significant and relevant changes in the PWS have been demonstrated for complex interventions including mindfulness-based programs [32]. The PWS was not included as an outcome measure in our previous pilot study [1] but based on the findings from the qualitative study of the WAYA program we have selected PWS as a primary outcome measure [26]. For the qualitative analysis of the pilot study, participants in the wilderness program were interviewed at 3 months after start of the program to explore the impact of the program on their health and well-being. Themes that arose through the inductive content analysis related to self-confidence, self-acceptance, meaningful relationships, and personal growth. These themes are also reflected within the domains of the PWS providing additional richness to the data [31].

2. Changes in nature connectedness of AYAs affected by cancer over time, measured via the Nature Relatedness Scale (NRS) [33]. The NRS is a 21-item scale that measures the ‘affective, cognitive, and physical relationship individuals’ have with the natural world. For the purpose of the previous pilot study [1], the NRS was translated and validated in the Swedish language (results not published).

Rationale: In line with guidance and instructions for RCTs on complex interventions [30], nature connectedness is hypothesized to be the underlying mechanism (intermediate outcome)

For peer review only - http://bmjopen.bmj.com/site/about/guidelines.xhtml

along the theorized impact path towards eudaemonic (psychological) well-being. A significant increase in NRS has previously been demonstrated in childhood and AYA cancer survivors after participation in the WAYA program compared to the hotel stay [1].

Secondary outcomes (exploratory)

Secondary outcomes explore possible changes in quality of life, physical activity, and fitness:

1. Changes in quality of life of AYAs affected by cancer over time, measured by the Minneapolis Manchester Quality of Life instrument (MMQL) [34]. The MMQL is a quality-of-life questionnaire specifically designed for cancer survivors and consists of seven quality of life domains: physical functioning, cognitive functioning, psychological functioning, body image, social functioning, intimate relations, and outlook on life. The instrument is translated and validated in the Swedish context.
2. Changes in light, and moderate-to-vigorous intensity physical activity will be measured objectively using ActiGraph [35].
3. Changes in maximal oxygen consumption (VO2max; ml/kg/min) will be estimated by means of the Ekblom Bak cycle ergometer test [36].
4. Blood Pressure and Heart Rate (BP/HR) will be assessed with an electronic monitor in a seated position after a 5-min rest. Two measurements will be performed, and the lowest value will be registered.
5. Body mass index (BMI) will be measured non-fasted and in light clothing to the nearest 0.1 kg by a digital scale. Height will be measured to the nearest 1 mm by a stadiometer. BMI (kg/m2) will be calculated.

Considering the large similarity between the Swedish and Norwegian language, the validated Swedish primary outcome scales (PWS, NRS, MMQL) will only be translated to the Norwegian language and back translated for quality control.

For peer review only - http://bmjopen.bmj.com/site/about/guidelines.xhtml

For peer review only

Safety-related outcomes

Occurrence of (serious) adverse effects during the interventions are reported and documented in the field diaries. Adverse effects will be coded according to the Medical Dictionary for Regulatory Activities (MedDRA) [37] and analyzed using preferred terms and allocation to system organ class. Safety of the programs will be evaluated by analyzing the number, seriousness, intensity, and types of adverse effects that are evaluated to be certain, probable/likely, or possibly related to the study programs. Equal safety between the wilderness program and hotel stay is defined as no significant differences in the risk of having serious adverse effects upon wilderness versus hotel stay participation.

Data collection

Table 1 below gives an overview of timepoints for data collection in the proposed study. The first part of this study has a follow-up time of one year. Additional to primary and secondary outcomes, other data will be collected for program comparison, evaluation, study quality and reporting (see Table 1). Lifestyle changes include diet, sleep, stress, physical activity (type, frequency/length), smoking, nicotine use, alcohol, caffeine, and outdoor/nature activities (type, frequency/length). Supportive relationships concern the experienced relationships with others affected by cancer (scale 1-5, not at all to very much). Self-efficacy outdoors concerns the confidence to spend time in nature/outdoors and their outdoor skills (scale 1-5, not at all to very much).

Table 1. Timeline of measurements in the study

**Measurements**	**Screening**	**Randomization**	**Intervention 1**		**Three-months at home**		**Intervention 2**	**One year**
	Contact 1	Contact 2	Day 1 **T=0***	Day 8* **T=1***	Contact 3	Contact 4	Month 3 **T=2***	Year 1 **T=3**
**Primary outcomes**								
PWS			X	X			X	X

For peer review only - http://bmjopen.bmj.com/site/about/guidelines.xhtml

NRS			X	X			X	X
**Secondary outcomes**								
MMQL			X	X			X	X
Physical activity			X	X			X	X
VO2max			X				X	
Blood pressure			X				X	
Heart rate			X				X	
Body mass index			X				X	
Adverse effects				X	X	X	X	X
**Other data**								
Willingness	X							
Preference	X							
Expectations	X							
Adherence				X			X	
Effectiveness		X			X	X		X
Demographic data	X							
Medical History		X						
Medication use		X			X	X		X
Other therapies/ self-care		X			X	X		X
Lifestyle changes		X			X	X		X
Supportive relationships		X			X	X		X
Self-efficacy outdoors		X			X	X		X
Outdoor activities		X			X	X		X
Physical activities		X			X	X		X

*To avoid possible effects of intervention-related expectations and anxiety and return-euphoria, all self-reported questionnaires will be filled in by participants 2-3 weeks before the start of the first intervention, 2-3 weeks after intervention, and 2-3 weeks after the second intervention. PWS: Psychological Well-being Scale, NRS: Nature Relatedness Scale, MMQL: Minneapolis Manchester Quality of Life instrument, VO2max: Maximal oxygen consumption. Willingness: participants' willingness to be randomized, Preference: participants' intervention preference, Expectations: participants' intervention expectations, Adherence: participants' intervention adherence, Effectiveness: participants' expected intervention effectiveness.

For peer review only

Sample size

The current study aims to recruit a total of 150 participants that will be randomized into two groups (n = 75 per group). The power calculation is based on a two-group linear mixed effects

For peer review only - http://bmjopen.bmj.com/site/about/guidelines.xhtml

model for repeated measures data with attrition [38] and provides 80% power to detect a medium-sized between-group difference (*SMD* = 0.60). In the sample size estimation, we account for an attrition rate of 34.5% before the start of the intervention as reported in the previous pilot study [1] and a low attrition rate (10%) during the intervention. The power calculation is based on a model with four repeated measurement points, a first-order autoregressive (AR) correlation structure, an initial repeated measures correlation of 0.60 (with decreasing magnitude of the repeated measures correlations with increasing distance between time points), an alpha level of .05, and a group allocation of 1:1.

The effect size estimates for the primary outcomes are based on a meta-analytic estimate [32] of a standardized between-group difference of 0.69 (Hedges' *g*) for interventions to enhance eudaemonic psychological well-being, and findings from a pilot study indicating a significant between-group difference in NRS between the wilderness program and hotel stay (Cohen's *d* = 0.63) [1].

Recruitment

Participants will be recruited among members of the Swedish cancer organizations Ung Cancer and the Childhood Cancer Fund, members of the Norwegian cancer organization Ung Kreft, and through oncology departments of hospitals in Norway and Sweden. In all news items, information and brochures, AYAs are directed to the website of the WAYA-2 study where they can register their interest for possible participation in the study [39].

Randomization and concealment

Participants will be randomized equally (1:1) to the wilderness program or hotel stay according to a randomization list as generated by a Random Allocation Software Program [40] using a random block size of two to guarantee a balanced allocation. Participants will be stratified according to two age groups (16-30, 31-39 years), binary gender categorizations (male/female), and cancer survivorship (childhood cancer: age 0-14 years versus AYA

For peer review only - http://bmjopen.bmj.com/site/about/guidelines.xhtml

For peer review only

cancer: age 15-39 years) to achieve equal distribution among the two groups. Per stratification and per country (Norway, Sweden), separate randomization lists will be generated. AYAs affected by cancer that have registered their interest in the study via the website will receive a screening number based on the date of registration entry. Eligible participants will be randomized and assigned to the wilderness program or hotel stay based on the fixed order of the screening numbers by a researcher that is not involved in the screening and intake of study participants. Allocation to program intervention will be concealed by the complexity of the randomization procedure involving a group of ten or more eligible participants at a time and randomizations over eight different strata during the period of one year.

Blinding

Upon screening, participants will be informed that two possibly effective programs will be investigated. To reduce expectation bias, it will not be made known to participants whether the wilderness program is hypothesized to be better than a hotel stay (single-blinded). Statistical analysis will be performed by a statistician that is blind to program allocation.

Data management and confidentiality

Study data of each participant will be handled in a case report form (CRF). Self-reported demographic data, medical data (cancer diagnosis, cancer treatment, medical history etc), and other relevant intervention-related data (preference, expectations, effectiveness etc) will also be documented in the CRF. Participants are allowed to continue all their medication, therapies and/or dietary and lifestyle measures during the study. Any changes in medication, therapies, dietary and lifestyle measures over time will be documented in the CRF. CRF data will be anonymised for analysis by assigning each participant an unidentifiable three-digit screening number at the time of enrolment and additional three-digit study number upon randomization. The CRF, SPSS dataset and other participant-related documentation will be stored on a secure server at Mid Sweden University or Agder University and can only be accessed with a

For peer review only - http://bmjopen.bmj.com/site/about/guidelines.xhtml

password by research team members that are authorized. To guarantee data quality, independent checks during data collection and data entry will be performed by another researcher than the one collecting or entering the data in the database. A data monitoring committee will not be established because of the nature of the study (health-promotion intervention with low risk). The collected data will be saved for at least ten years after the end of the study.

Data analysis

Chi-square statistics, independent samples t-tests, and analysis of variance (ANOVA) will be used to calculate group differences in socio-demographic variables and cancer characteristics at baseline. The Alpha level will be set to 0.05. The primary analysis will investigate changes in the total scores of the PWS, MMQL and NRS over time from baseline to 1-year follow-up in the intervention group compared to the control group. Missing data will be imputed by means of multiple imputation or by using full-information maximum likelihood estimation [41]. The analysis of the primary outcome will be based on a linear mixed effects model and will be fitted to test intervention effects up to 1 year. Adjustments will be made for age, gender, age at cancer diagnoses, and study site. Time, intervention arm, interaction between time and intervention arm, age, gender, age at cancer diagnoses, and study site are included as fixed effects, and participant is included as random effect in the model. Statistical analyses will be on the intention-to-treat (ITT) principle (all participants that are randomized in the study). As sensitivity analyses, the per-protocol data set will be analysed including only participants that have participated for at least one day in the wilderness or hotel stay. Secondary outcomes will also be analysed using linear mixed effects models.

Categorical data will be presented as frequencies and percentages. For continuous data, the number, mean, standard deviation, median, inter-quartile range, minimum and maximum will be calculated. A two-sided p-value <0.05 will be considered statistically significant.

For peer review only

Safety analyses will be performed on the safety analysis set and include all individuals that had participated in one of the program interventions for at least one day. Relative Risk (RR) with 95% Confidence Intervals (CI) will be calculated to indicate the risk of having an adverse effect adjusted to days of exposure in program interventions. Pearson's chi-squared test will be used to calculate differences in adverse effects severity between the two study arms. A detailed description of the planned statistical analyses will be provided in the Statistical Analysis Plan (SAP), which will be finalized prior to database closure and data analysis. All statistical analysis will be performed using Stata version 18.0.

Protocol amendments

Possible changes to this protocol, and reasons for these changes, will be documented in the trial register (www.isrctn.com), and if needed submitted to the Swedish and Norwegian Ethical Review Authority for approval. The registered study protocol (ISRCTN93856392) includes all items on the minimum standard check list of items of the World Health Organization [42].

Ethics and dissemination

Participants are informed both orally and in writing that participation in the WAYA-2 study is voluntary. It is made clear that participation in all study-related activities is voluntary, that they can decline participation in any activity without explanation, and that they can withdraw at any time from the program without stating a reason. Participants are further informed about the purpose of the study, that data will be handled and later published and presented in a confidential way. Informed consent will be obtained from all participants in writing prior to the start of the study and send to the researcher responsible for screening and intake. Participation in the WAYA program is without costs for participants.

For peer review only - http://bmjopen.bmj.com/site/about/guidelines.xhtml

The WAYA-2 study will be performed in line with the instructions and precautions as described in the study safety plan [2]. A physician with emergency medicine expertise will be available by telephone in case of emergency. Adequate insurance for all participants and facilitators in the study will be provided by Mid Sweden University or Agder University. The study protocol was approved by the Swedish Ethical Review Authority on 27 September 2023 (reference: 2023-05247-01). The study application to the Norwegian Ethical Review Authority is pending (reference: 694114). Information letters and consent forms will be accessible upon request.

We aim for a wide communication and dissemination of research results through close collaboration with national and international research partners and the wider communities in the respective countries. Results of the study will be presented to the participants. It is aimed to publish at least three scientific (peer-reviewed) articles and to present the results at (scientific) national and international conferences. Popular articles with study results will be disseminated in the form of interviews, books, and chronicles. News items will also be spread via social media (Facebook, Twitter), websites and newsletters of collaborating partners and organizations and of the funding bodies.

Patient and Public Involvement

User involvement and collaboration with the wider community was established during our previous WAYA-1 study and will be continued in the present study. Three AYAs affected by cancer are in the advisory board of the WAYA-2 study. For all wilderness and hotel stay interventions, at least one member of the facilitating team will be an AYA affected by cancer. The WAYA-2 study design and recruitment strategies have been developed in close collaboration with AYAs affected by cancer, the Swedish cancer organizations for childhood and young cancer, researchers from four different countries (Netherlands, Norway, Sweden, USA), and health care professionals from the region Västernorrland in Sweden. The content

For peer review only - http://bmjopen.bmj.com/site/about/guidelines.xhtml

of the WAYA-2 program and hotel stay has been developed in close collaboration with the Swedish survival guilt, other outdoor instructors/nature guides, and the Swedish cancer organization and optimized through feedback from previous participants in the WAYA-1 study. All parties as mentioned above will be involved in interpretation and dissemination of study results.

Timeline of the study

The timeline of the study is depicted in Figure 1. The first participants were enrolled in the study on 19 February 2024. The first interventions in Sweden will be carried out during the summer of 2024. The interventions in Norway will take place in the summer of 2025. Since participants are followed for one year after the start of the intervention, data collection will end in July 2026. Data analyses are planned to start in December 2026 and reporting is planned to end in December 2027. It is the aim of this study that participants will be followed up for ten years after the study start (two-, five- and ten-year follow-up) to investigate possible long-term changes in psychological well-being, health related quality of life and physical activity. Ethical approval and finance for the long-term follow-up of the WAYA-2 study will be sought in 2025.

DISCUSSION

AYAs affected by cancer are an understudied and vulnerable group and effective interventions are needed to ensure that they are able to cope with late effects of cancer, its treatment, and achieve good quality of life. To the best of our knowledge, worldwide this study will be the first full-scale RCT that investigates the effectiveness of a wilderness program for AYAs affected by cancer, and the first wilderness program in Scandinavia that is developed and adapted to their needs. The WAYA program in this study is specifically

For peer review only - http://bmjopen.bmj.com/site/about/guidelines.xhtml

designed to offer a supervised wilderness program to AYAs with a wide range of disabilities [2].

This study aims to demonstrate the evidence base for a wilderness program intending to impact the psychological well-being of AYAs affected by cancer. Expected short-term impact is that the wilderness program may support their self-efficacy, self-confidence, and provide them with the competence to develop and maintain their own outdoor practice. The long-term impact of the wilderness program may decrease depression, anxiety, and stress, and increase quality of life. In the development of the previous pilot RCT design, considerable effort was invested to define an appropriate control group activity [23]. A hotel stay was chosen to control for attention from facilitators, group support, and being away from their home environment. Although it might be expected that relaxation and connecting with other AYAs offer positive experiences and time to recharge, it is expected that these may have less impact on their health and well-being in the long-term [43, 44]. Based on previous experiences with the feasibility of a planned RCT in Norway [45], it was decided not to include a "care as usual" or wait-list control arm in the WAYA-2 study.

This study is expected to fill a gap in the literature by providing valuable insights and needed evidence on the role of nature in the support of the health and well-being of such a vulnerable and understudied population. This will inform AYAs affected by cancer, their families, outdoor instructors, clinicians, and other researchers.

One of the expected challenges of the present study is to recruit the needed number of participants in the study within the set timeframe. We previously published that the eligibility (87,3%) and enrolment rate (95.2%) of participants in the WAYA-1 study were high [21]. To randomize 150 eligible individuals into the WAYA-2 study, we therefore need to recruit at least 180 AYAs affected by cancer to register their interest to participate in the WAYA-2

For peer review only - http://bmjopen.bmj.com/site/about/guidelines.xhtml

study. In our previous pilot study we only recruited among members of Swedish cancer organizations Ung Cancer and the Childhood Cancer Fund, and the recruitment rate was low (3.6%) [21]. However, the pilot study was performed during the COVID-19 pandemic where recommendations with respect to travel restrictions were in place. It is therefore expected a higher recruitment rate in the WAYA-2 study. Furthermore, we will now also recruit participants in Norway and additional recruitment strategies will be developed with support of healthcare professionals and brochures in waiting rooms of oncology departments of hospitals in Norway and Sweden.

Another potential challenge of the WAYA-2 study is the inclusion of a heterogenous study population (all cancer diagnosis and all cancer treatment stages) with a wide age range (16-39 years). Program activities and study outcomes may be affected by participants being at very different stages of their cancer trajectory and/or lives. The inclusion of participants with a large span was investigated in detail in the previous pilot study and results have been published [2]. It appeared that the wide age range and different stages in the cancer process was perceived to be an advantage (rather than a disadvantage), since it positively influenced group bonding and learning process, according to facilitators and participants.

This study involves a unique cross-sectional international (Norway, Sweden, USA) and multidisciplinary collaboration between academic researchers, clinicians, outdoor instructors, public health professionals, patient organizations, and non-profit organizations in the outdoor sector. Members of the research team have expertise in clinical trials, epidemiology, nature-based therapies, nursing, oncology, psycho-oncology, public health, social sciences, sport sciences, wilderness therapy, and have previously published on the topic [1, 2, 22, 23, 46-49]. It is expected that such a collaboration is needed to tackle the complexity of this multi-component intervention for AYAs affected by cancer.

For peer review only - http://bmjopen.bmj.com/site/about/guidelines.xhtml

STUDY STATUS

First participants were enrolled in the study on 19 February 2024. The estimated end of the first part of the study (one year follow-up) is planned on 31 December 2027.

ACKNOWLEDGEMENTS

The authors thank Jens Annamatz Ranvide, Susanne Johansson, and Elvira Rolandsdotter for their valuable comments, advice, and contributions to the WAYA-2 study. Sandra Häggander of Ung Cancer Sweden, Sara Hellberg and Bea Eriksson from the Swedish Childhood Cancer Fund (Maxa livet) are thanked for their constructive input and feedback related to the performance of the study.

AUTHOR CONTRIBUTIONS

EAL, MCJ, and MJ conceived the idea for the study. MJ is the principal investigator in Sweden and SB is the principal investigator in Norway. MCJ will coordinate the study in Sweden and CRF will coordinate the study in Norway. The WAYA-2 study is designed by all co-authors. MCJ drafted the first version of the protocol and manuscript. AS will perform the statistical analyses. AS, CRF, DEV, EAL, MJ and SB critically revised the study protocol and manuscript. All authors approved of the manuscript. Regarding publication of results, authorship eligibility guidelines of the respective peer-reviewed scientific journals will be followed.

FUNDING STATEMENT

Research grants have been obtained from Ekhagastiftelsen (grant no 2023-29) and Sjöbergstiftelsen (grant no 2024-01-04:10) in Sweden. Funders have no possibility to influence the study design, data collection, management, and analysis, study results, writing and publication of scientific articles. In-kind financial contributions to the study will be provided for by Mid Sweden University (Sundsvall, Sweden), the National Research Center

For peer review only - http://bmjopen.bmj.com/site/about/guidelines.xhtml

in Complementary and Alternative Medicine (NAFKAM) (Tromsø, Norway), and the University of Agder (Kristiansand, Norway).

DATA AVAILABILITY STATEMENT

The final dataset from this ongoing study will become available after publication of the results upon request to the corresponding author.

COMPETING INTERESTS STATEMENT

None declared

Figure 1. Time line of the WAYA-2 study

REFERENCES

1. Jong MC, Dahlqvist H, Lown EA, et al. A randomized controlled pilot study assessing feasibility and safety of a wilderness program for childhood, adolescent, and young adult cancer survivors: the WAYA study. *BMC Public Health* 2023;**23**(1):1504. doi: 10.1186/s12889-023-16408-x
2. Jong MC, Stub T, Mulder E, et al. The Development and Acceptability of a Wilderness Programme to Support the Health and Well-Being of Adolescent and Young Adult Cancer Survivors: The WAYA Programme. *Int J Environ Res Public Health* 2022;**19**(19):12012.
3. Murphy CC, Lupo PJ, Roth ME, et al. Disparities in Cancer Survival Among Adolescents and Young Adults: A Population-Based Study of 88 000 Patients. *J Natl Cancer Inst* 2021;**113**(8):1074-83. doi: 10.1093/jnci/djab006 [published Online First: 2021/01/24]
4. Mellblom AV, Kiserud CE, Rueegg CS, et al. Self-reported late effects and long-term follow-up care among 1889 long-term Norwegian Childhood, Adolescent, and Young Adult Cancer Survivors (the NOR-CAYACS study). *Support Care Cancer* 2021;**29**(6):2947-57. doi: 10.1007/s00520-020-05790-6

For peer review only - http://bmjopen.bmj.com/site/about/guidelines.xhtml

5. Oeffinger KC, Mertens AC, Sklar CA, et al. Chronic health conditions in adult survivors of childhood cancer. *N Engl J Med* 2006;**355**(15):1572-82. doi: 10.1056/NEJMsa060185 [published Online First: 2006/10/13]
6. Larsen MH, Larsen EH, Ruud E, et al. "I have to do things differently now, but I make it work"—Young childhood cancer survivors' experiences of self-management in everyday living. *J Cancer Surviv* 2022;**16**(4):728-40. doi: 10.1007/s11764-021-01066-y
7. Crowder SL, Sauls R, Gudenkauf LM, et al. The Lived Experience of Young Adult Cancer Survivors after Treatment: A Qualitative Study. *Nutrients* 2023;**15**(14):3145.
8. Cheung AT, Li WHC, Ho LLK, et al. Physical activity for pediatric cancer survivors: a systematic review of randomized controlled trials. *J Cancer Surviv* 2021;**15**(6):876-89. doi: 10.1007/s11764-020-00981-w [published Online First: 2021/01/04]
9. Moberg L, Fritch J, Westmark D, et al. Effect of physical activity on fatigue in childhood cancer survivors: a systematic review. *Support Care Cancer* 2022;**30**(8):6441-49. doi: 10.1007/s00520-022-06960-4
10. Wogksch MD, Goodenough CG, Finch ER, et al. Physical activity and fitness in childhood cancer survivors: A scoping review. *Aging and Cancer* 2021;**2**(4):112-28. doi: https://doi.org/10.1002/aac2.12042
11. Zhi X, Xie M, Zeng Y, et al. Effects of Exercise Intervention on Quality of Life in Adolescent and Young Adult Cancer Patients and Survivors: A Meta-Analysis. *Integr Cancer Ther* 2019;**18**:1534735419895590. doi: 10.1177/1534735419895590 [published Online First: 2019/12/18]
12. Adams SC, Petrella A, Sabiston CM, et al. Preferences for exercise and physical activity support in adolescent and young adult cancer survivors: a cross-sectional survey. *Support Care Cancer* 2021;**29**(7):4113-27. doi: 10.1007/s00520-020-05897-w
13. Cox CL, Montgomery M, Oeffinger KC, et al. Promoting physical activity in childhood cancer survivors: results from the Childhood Cancer Survivor Study. *Cancer* 2009;**115**(3):642-54. doi: 10.1002/cncr.24043 [published Online First: 2009/01/02]
14. Xu FJ ZL, Miao BH, Wang BJ, Li H, Chen Y, Wang HM,. Research progress on physical activity of childhood cancer survivors. *Chinese journal of preventive medicine* 2024;**58**(2):177-82.
15. Ahmadi F, Ahmadi N. Nature as the Most Important Coping Strategy Among Cancer Patients: A Swedish Survey. *J Relig Health* 2015;**54**(4):1177-90. doi: 10.1007/s10943-013-9810-2 [published Online First: 2013/12/24]

For peer review only - http://bmjopen.bmj.com/site/about/guidelines.xhtml

16. Blaschke S. The role of nature in cancer patients' lives: a systematic review and qualitative meta-synthesis. *BMC Cancer* 2017;**17**(1):370. doi: 10.1186/s12885-017-3366-6 [published Online First: 2017/05/27]

17. Blaschke S, O'Callaghan CC, Schofield P. Cancer Patients' Recommendations for Nature-Based Design and Engagement in Oncology Contexts: Qualitative Research. *HERD: Health Environments Research & Design Journal* 2017;**11**(2):45-55. doi: 10.1177/1937586717737813

18. Timko Olson ER, Olson AA, Driscoll M, et al. Nature-Based Interventions and Exposure among Cancer Survivors: A Scoping Review. *Int J Environ Res Public Health* 2023;**20**(3) doi: 10.3390/ijerph20032376 [published Online First: 2023/02/12]

19. Lesser IA, Nienhuis CP, Belanger L. Active by nature: exploring cancer survivors' exercise barriers, facilitators, preferences, and psychosocial benefits of engaging in outdoor physical activity. *Support Care Cancer* 2021;**29**(7):4095-103. doi: 10.1007/s00520-020-05938-4

20. Park EY, An MY, Sung JH. A present from the forest: Focus group interviews on cancer survivors' forest experiences. *Asia Pac J Oncol Nurs* 2022;**9**(8):100105. doi: 10.1016/j.apjon.2022.100105 [published Online First: 2022/09/06]

21. de Vries S, Verheij RA, Groenewegen PP, et al. Natural Environments—Healthy Environments? An Exploratory Analysis of the Relationship between Greenspace and Health. *Environment and Planning A: Economy and Space* 2003;**35**(10):1717-31. doi: 10.1068/a35111

22. Jong M, Lown EA, Schats W, et al. A scoping review to map the concept, content, and outcome of wilderness programs for childhood cancer survivors. *PLOS ONE* 2021;**16**(1):e0243908. doi: 10.1371/journal.pone.0243908

23. Jong MC, Mulder E, Kristoffersen AE, et al. Protocol of a mixed-method randomised controlled pilot study evaluating a wilderness programme for adolescent and young adult cancer survivors: the WAYA study. *BMJ Open* 2022;**12**(5):e061502. doi: 10.1136/bmjopen-2022-061502

24. Naess A. Self-realization: An ecological approach to being in the world. *Trumpeteer* 1987;**4**:35-42.

25. Huber M, van Vliet M, Giezenberg M, et al. Towards a 'patient-centred' operationalisation of the new dynamic concept of health: a mixed methods study. *BMJ Open* 2016;**6**(1):e010091. doi: 10.1136/bmjopen-2015-010091

26. Jong M, Stub T, Jong MC. Supporting Personal Growth in Childhood, Adolescent and Young-Adult Cancer Survivors Through Challenges in Nature — A Qualitative Study of WAYA Wilderness Programme Participation. *Global Advances in Integrative*

For peer review only - http://bmjopen.bmj.com/site/about/guidelines.xhtml

Medicine and Health 2024;**13**:27536130241238150. doi: 10.1177/27536130241238150
27. Chan A-W, Tetzlaff JM, Gøtzsche PC, et al. SPIRIT 2013 explanation and elaboration: guidance for protocols of clinical trials. *BMJ : British Medical Journal* 2013;**346**:e7586. doi: 10.1136/bmj.e7586
28. CONSORT 2010 Statement: Updated Guidelines for Reporting Parallel Group Randomized Trials. *Ann Intern Med* 2010;**152**(11):726-32. doi: 10.7326/0003-4819-152-11-201006010-00232 %m 20335313
29. Naess A. Ecology, community and lifestyle. Cambridge: Cambridge University Press 1990.
30. Leroy JL, Frongillo EA, Kase BE, et al. Strengthening causal inference from randomised controlled trials of complex interventions. *BMJ Glob Health* 2022;**7**(6) doi: 10.1136/bmjgh-2022-008597 [published Online First: 2022/06/11]
31. Ryff CD. Happiness is everything, or is it? Explorations on the meaning of psychological well-being. *J Pers Soc Psychol* 1989;**57**(6):1069-81. doi: 10.1037/0022-3514.57.6.1069
32. van Dierendonck D, Lam H. Interventions to enhance eudaemonic psychological well-being: A meta-analytic review with Ryff's Scales of Psychological Well-being. *Applied Psychology: Health and Well-Being* 2023;**15**(2):594-610. doi: https://doi.org/10.1111/aphw.12398
33. Nisbet EK, Zelenski JM, Murphy SA. The Nature Relatedness Scale:Linking Individuals' Connection With Nature to Environmental Concern and Behavior. *Environment and Behavior* 2009;**41**(5):715-40. doi: 10.1177/0013916508318748
34. Bosworth A, Goodman EL, Wu E, et al. The Minneapolis-Manchester Quality of Life Instrument: reliability and validity of the Adult Form in cancer survivors. *Qual Life Res* 2018;**27**(2):321-32. doi: 10.1007/s11136-017-1671-9 [published Online First: 2017/08/05]
35. Berntsen S, Hageberg R, Aandstad A, et al. Validity of physical activity monitors in adults participating in free-living activities. *Br J Sports Med* 2010;**44**(9):657-64. doi: 10.1136/bjsm.2008.048868 [published Online First: 2008/07/17]
36. Björkman F, Ekblom-Bak E, Ekblom Ö, et al. Validity of the revised Ekblom Bak cycle ergometer test in adults. *Eur J Appl Physiol* 2016;**116**(9):1627-38. doi: 10.1007/s00421-016-3412-0 [published Online First: 2016/06/18]
37. Medical Dictionary for Regulatory Activities (MedDRA®)2007.

For peer review only - http://bmjopen.bmj.com/site/about/guidelines.xhtml

38. Lu K, Luo X, Chen PY. Sample size estimation for repeated measures analysis in randomized clinical trials with missing data. *Int J Biostat* 2008;**4**(1):Article 9. doi: 10.2202/1557-4679.1098 [published Online First: 2008/01/01]

39. M id Sweden University. WAYA-2 study 2024 [Available from: https://www.miun.se/waya-anmalan accessed 1 March 2024.

40. Ltd. SE. Create a blocked randomisation list 2022 [Available from: https://www.sealedenvelope.com accessed 12 February 2024.

41. Enders C. Missing Data: An Update on the State of the Art. *Psychol Methods* 2023 doi: 10.1037/met0000563

42. WHO. WHO Trial Registration Data Set [Version 1.3.1:[Available from: https://www.who.int/clinical-trials-registry-platform/network/who-data-set accessed 29 February 2024.

43. de Bloom J, Geurts SAE, Taris TW, et al. Effects of vacation from work on health and well-being: Lots of fun, quickly gone. *Work Stress* 2010;**24**(2):196-216. doi: 10.1080/02678373.2010.493385

44. de Bloom J, Kompier M, Geurts S, et al. Do we recover from vacation? Meta-analysis of vacation effects on health and well-being. *J Occup Health* 2009;**51**(1):13-25. doi: 10.1539/joh.k8004 [published Online First: 2008/12/20]

45. Gabrielsen LE, Fernee CR, Aasen GO, et al. Why Randomized Trials Are Challenging Within Adventure Therapy Research:Lessons Learned in Norway. *J Exper Edu* 2016;**39**(1):5-14. doi: 10.1177/1053825915607535

46. Harper NJ, Fernee CR, Gabrielsen LE. Nature's Role in Outdoor Therapies: An Umbrella Review. *Int J Environ Res Public Health* 2021;**18**(10) doi: 10.3390/ijerph18105117 [published Online First: 2021/06/03]

47. Lown EA, Otto HR, Norton CL, et al. Program evaluation of a wilderness experience for adolescents facing cancer: A time in nature to heal, connect and find strength. *PLOS ONE* 2023;**18**(10):e0291856. doi: 10.1371/journal.pone.0291856

48. Stea TH, Jong MC, Fegran L, et al. Mapping the Concept, Content, and Outcome of Family-Based Outdoor Therapy for Children and Adolescents with Mental Health Problems: A Scoping Review. *Int J Environ Res Public Health* 2022;**19**(10):5825.

49. Victorson D, Doninger G, Victorson S, et al. Psychosocial and Biological Outcomes of Immersive, Mindfulness-Based Treks in Nature for Groups of Young Adults and Caregivers Affected by Cancer: Results from a Single Arm Program Evaluation from 2016-2021. *Int J Environ Res Public Health* 2021;**18**(23) doi: 10.3390/ijerph182312622 [published Online First: 2021/12/11]

For peer review only

For peer review only - http://bmjopen.bmj.com/site/about/guidelines.xhtml

For peer review only

For peer review only - http://bmjopen.bmj.com/site/about/guidelines.xhtml

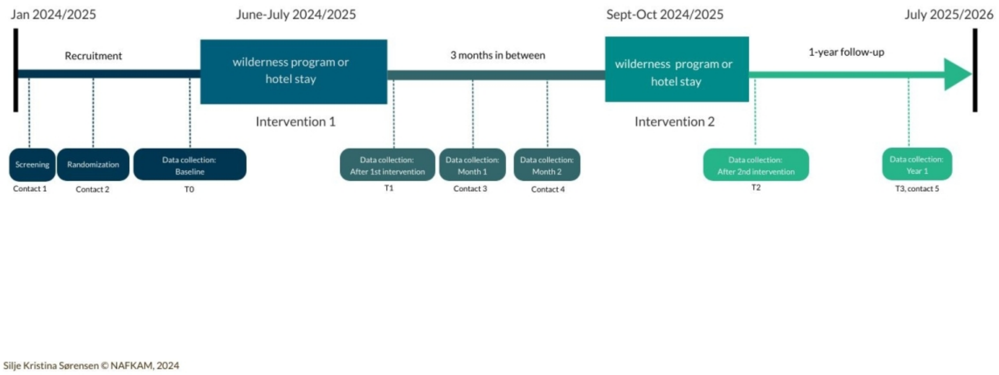

Time line of the WAYA-2 study

540x297mm (72 x 72 DPI)

STANDARD PROTOCOL ITEMS: RECOMMENDATIONS FOR INTERVENTIONAL TRIALS

SPIRIT 2013 Checklist: Recommended items to address in a clinical trial protocol and related documents*

Section/item	Item No	Description	Addressed on page number
**Administrative information**			
Title	1	Descriptive title identifying the study design, population, interventions, and, if applicable, trial acronym	____1_________
Trial registration	2a	Trial identifier and registry name. If not yet registered, name of intended registry	____3_______
	2b	All items from the World Health Organization Trial Registration Data Set	____18________
Protocol version	3	Date and version identifier	____2_________
Funding	4	Sources and types of financial, material, and other support	____23________
Roles and responsibilities	5a	Names, affiliations, and roles of protocol contributors	____1, 23_____
	5b	Name and contact information for the trial sponsor	_____NA______
	5c	Role of study sponsor and funders, if any, in study design; collection, management, analysis, and interpretation of data; writing of the report; and the decision to submit the report for publication, including whether they will have ultimate authority over any of these activities	_____23_____
	5d	Composition, roles, and responsibilities of the coordinating centre, steering committee, endpoint adjudication committee, data management team, and other individuals or groups overseeing the trial, if applicable (see Item 21a for data monitoring committee)	_____NA______

For peer review only

For peer review only - http://bmjopen.bmj.com/site/about/guidelines.xhtml

**Introduction**			
Background and rationale	6a	Description of research question and justification for undertaking the trial, including summary of relevant studies (published and unpublished) examining benefits and harms for each intervention	____4-6, 7___
	6b	Explanation for choice of comparators	_____7, 21_____
Objectives	7	Specific objectives or hypotheses	______7_____
Trial design	8	Description of trial design including type of trial (eg, parallel group, crossover, factorial, single group), allocation ratio, and framework (eg, superiority, equivalence, noninferiority, exploratory)	______7______
**Methods: Participants, interventions, and outcomes**			
Study setting	9	Description of study settings (eg, community clinic, academic hospital) and list of countries where data will be collected. Reference to where list of study sites can be obtained	____ 7, 9__
Eligibility criteria	10	Inclusion and exclusion criteria for participants. If applicable, eligibility criteria for study centres and individuals who will perform the interventions (eg, surgeons, psychotherapists)	______8____
Interventions	11a	Interventions for each group with sufficient detail to allow replication, including how and when they will be administered	______8-9____
	11b	Criteria for discontinuing or modifying allocated interventions for a given trial participant (eg, drug dose change in response to harms, participant request, or improving/worsening disease)	______18____
	11c	Strategies to improve adherence to intervention protocols, and any procedures for monitoring adherence (eg, drug tablet return, laboratory tests)	______NA____
	11d	Relevant concomitant care and interventions that are permitted or prohibited during the trial	_____ 16____
Outcomes	12	Primary, secondary, and other outcomes, including the specific measurement variable (eg, systolic blood pressure), analysis metric (eg, change from baseline, final value, time to event), method of aggregation (eg, median, proportion), and time point for each outcome. Explanation of the clinical relevance of chosen efficacy and harm outcomes is strongly recommended	_____11-14___
Participant timeline	13	Time schedule of enrolment, interventions (including any run-ins and washouts), assessments, and visits for participants. A schematic diagram is highly recommended (see Figure)	__13-14, Figure 1

Sample size	14	Estimated number of participants needed to achieve study objectives and how it was determined, including clinical and statistical assumptions supporting any sample size calculations	_____15_____
Recruitment	15	Strategies for achieving adequate participant enrolment to reach target sample size	_____15, 22____

Methods: Assignment of interventions (for controlled trials)

Allocation:

Sequence generation	16a	Method of generating the allocation sequence (eg, computer-generated random numbers), and list of any factors for stratification. To reduce predictability of a random sequence, details of any planned restriction (eg, blocking) should be provided in a separate document that is unavailable to those who enrol participants or assign interventions	_____15-16____
Allocation concealment mechanism	16b	Mechanism of implementing the allocation sequence (eg, central telephone; sequentially numbered, opaque, sealed envelopes), describing any steps to conceal the sequence until interventions are assigned	_____15-16___
Implementation	16c	Who will generate the allocation sequence, who will enrol participants, and who will assign participants to interventions	_____15-16____
Blinding (masking)	17a	Who will be blinded after assignment to interventions (eg, trial participants, care providers, outcome assessors, data analysts), and how	_____15-16___
	17b	If blinded, circumstances under which unblinding is permissible, and procedure for revealing a participant's allocated intervention during the trial	______NA___

Methods: Data collection, management, and analysis

Data collection methods	18a	Plans for assessment and collection of outcome, baseline, and other trial data, including any related processes to promote data quality (eg, duplicate measurements, training of assessors) and a description of study instruments (eg, questionnaires, laboratory tests) along with their reliability and validity, if known. Reference to where data collection forms can be found, if not in the protocol	_____16-17____
	18b	Plans to promote participant retention and complete follow-up, including list of any outcome data to be collected for participants who discontinue or deviate from intervention protocols	_____16-17___

Data management	19	Plans for data entry, coding, security, and storage, including any related processes to promote data quality (eg, double data entry; range checks for data values). Reference to where details of data management procedures can be found, if not in the protocol	____16-17____
Statistical methods	20a	Statistical methods for analysing primary and secondary outcomes. Reference to where other details of the statistical analysis plan can be found, if not in the protocol	_____17-18____
	20b	Methods for any additional analyses (eg, subgroup and adjusted analyses)	____ 17-18___
	20c	Definition of analysis population relating to protocol non-adherence (eg, as randomised analysis), and any statistical methods to handle missing data (eg, multiple imputation)	_____17-18____
**Methods: Monitoring**			
Data monitoring	21a	Composition of data monitoring committee (DMC); summary of its role and reporting structure; statement of whether it is independent from the sponsor and competing interests; and reference to where further details about its charter can be found, if not in the protocol. Alternatively, an explanation of why a DMC is not needed	_____17____
	21b	Description of any interim analyses and stopping guidelines, including who will have access to these interim results and make the final decision to terminate the trial	______NA____
Harms	22	Plans for collecting, assessing, reporting, and managing solicited and spontaneously reported adverse events and other unintended effects of trial interventions or trial conduct	______13_____
Auditing	23	Frequency and procedures for auditing trial conduct, if any, and whether the process will be independent from investigators and the sponsor	______NA____
**Ethics and dissemination**			
Research ethics approval	24	Plans for seeking research ethics committee/institutional review board (REC/IRB) approval	______19_____
Protocol amendments	25	Plans for communicating important protocol modifications (eg, changes to eligibility criteria, outcomes, analyses) to relevant parties (eg, investigators, REC/IRBs, trial participants, trial registries, journals, regulators)	______18_____

Consent or assent	26a	Who will obtain informed consent or assent from potential trial participants or authorised surrogates, and how (see Item 32)	_____18-19____
	26b	Additional consent provisions for collection and use of participant data and biological specimens in ancillary studies, if applicable	_______NA____
Confidentiality	27	How personal information about potential and enrolled participants will be collected, shared, and maintained in order to protect confidentiality before, during, and after the trial	_____16-17_____
Declaration of interests	28	Financial and other competing interests for principal investigators for the overall trial and each study site	_____24_____
Access to data	29	Statement of who will have access to the final trial dataset, and disclosure of contractual agreements that limit such access for investigators	_______16-17___
Ancillary and post-trial care	30	Provisions, if any, for ancillary and post-trial care, and for compensation to those who suffer harm from trial participation	_____19_____
Dissemination policy	31a	Plans for investigators and sponsor to communicate trial results to participants, healthcare professionals, the public, and other relevant groups (eg, via publication, reporting in results databases, or other data sharing arrangements), including any publication restrictions	_____19________
	31b	Authorship eligibility guidelines and any intended use of professional writers	____23_______
	31c	Plans, if any, for granting public access to the full protocol, participant-level dataset, and statistical code	___ 24________
**Appendices**			
Informed consent materials	32	Model consent form and other related documentation given to participants and authorised surrogates	_____19______
Biological specimens	33	Plans for collection, laboratory evaluation, and storage of biological specimens for genetic or molecular analysis in the current trial and for future use in ancillary studies, if applicable	_____NA______

*It is strongly recommended that this checklist be read in conjunction with the SPIRIT 2013 Explanation & Elaboration for important clarification on the items. Amendments to the protocol should be tracked and dated. The SPIRIT checklist is copyrighted by the SPIRIT Group under the Creative Commons "Attribution-NonCommercial-NoDerivs 3.0 Unported" license.

For peer review only - http://bmjopen.bmj.com/site/about/guidelines.xhtml

BMJ Open

Protocol of a randomized controlled multi-centre trial investigating the effectiveness and safety of a wilderness program on the mental and physical well-being of adolescents and young adults affected by cancer: the WAYA-2 study.

Journal:	*BMJ Open*
Manuscript ID	bmjopen-2024-087626.R1
Article Type:	Protocol
Date Submitted by the Author:	08-May-2024

Complete List of Authors:	Jong, Miek; Mid Sweden University, Department of Health Sciences; UiT The Arctic University of Norway Faculty of Health Sciences, Department of Community Medicine, National Research Center in Complementary and Alternative Medicine (NAFKAM)<br>Fernee, Carina Ribe; Sorlandet Hospital Kristiansand, Department of Child and Adolescent Mental Health; University of Agder, Department of Sport Science and Physical Education<br>Stenling, Andreas; University of Agder, Department of Sport Science and Physical Education; Umeå University, Department of Psychology<br>Lown, E. Anne; University of California San Francisco, Department of Social & Behavioral Sciences<br>Berntsen, Sveinung; University of Agder, Department of Sport Science and Physical Education<br>Victorson , David; Northwestern University - Chicago Campus, Department of Medical Social Sciences, Feinberg School of Medicine<br>Jong, Mats; Mid Sweden University, Health Sciences

**Primary Subject Heading**:	Oncology
Secondary Subject Heading:	Public health
Keywords:	Safety, Quality of Life, ONCOLOGY, Psychosocial Intervention, Self-Management

SCHOLARONE™ Manuscripts

Protocol of a randomized controlled multi-centre trial investigating the effectiveness and safety of a wilderness program on the mental and physical well-being of adolescents and young adults affected by cancer: the WAYA-2 study.

Miek C. Jong[1,2], Carina Ribe Fernee[3,4], Sveinung Berntsen[4], Andreas Stenling[4,5], David E Victorson[6], E. Anne Lown[7], Mats Jong[1]

[1]Mid Sweden University, Department of Health Sciences, Sundsvall, Sweden.

[2]The Arctic University of Norway, National Research Center in Complementary and Alternative Medicine (NAFKAM), Department of Community Medicine, Faculty of Health Sciences, UiT, Tromsø, Norway.

[3]Department of Child and Adolescent Mental Health, Sorlandet Hospital, Kristiansand, Norway.

[4]Department of Sport Science and Physical Education, University of Agder, Kristiansand, Norway.

[5]Department of Psychology, Umeå University, Umeå, Sweden.

[6]Department of Medical Social Sciences, Feinberg School of Medicine, Northwestern University, Chicago, IL, USA.

[7]Department of Social & Behavioral Sciences, University of California San Francisco, San Francisco, USA.

Correspondence to:

Miek C. Jong, The Arctic University of Norway, National Research Center in Complementary and Alternative Medicine (NAFKAM), Department of Community Medicine, Faculty of Health Sciences, UiT, Hansine Hansens veg 18, 9019 Tromsø, Norway.

T: +46 10 142 89 67, email address: miek.jong@uit.no

For peer review only - http://bmjopen.bmj.com/site/about/guidelines.xhtml

Keywords: Oncology, psychosocial intervention, safety, self-management, quality of life

Word count: 5242

Date/version: 07-05-2024, version 7

ABSTRACT (max 300 words): 297

Introduction

Adolescent and young adults (AYAs) affected by cancer are an understudied group. Effective interventions are needed to support coping with late effects of cancer, its treatment, and to promote quality of life. Nature-based interventions may be promising in support of the self-management and health of AYAs affected by cancer. However, randomized controlled studies (RCTs) on the effectiveness of such interventions are lacking. We performed a first pilot RCT (n=42) that showed that it is feasible and safe to conduct such a study. Here we propose a full scale RCT to investigate the effectiveness and safety of a wilderness program on the mental and physical health of AYAs affected by cancer.

Methods and analysis

Participants are 150 AYAs affected by cancer, aged 16-39 years, who will be randomized to a wilderness (n=75) or a hotel stay (n=75). The wilderness program is an eight-day intervention including a six-day wilderness expedition. This is followed three months later by a four-day intervention including a two-day basecamp. Activities include hiking, backpacking, kayaking, rock climbing, mindfulness, and bush-crafting. The comparison group is an eight-day hotel stay followed by a four-day hotel stay (interventions include two travel days) at the same hotel after three months. Primary outcomes are psychological well-being and nature connectedness up to one year after study start. Secondary outcomes are quality of life, physical activity, and safety parameters.

For peer review only - http://bmjopen.bmj.com/site/about/guidelines.xhtml

For peer review only

Ethics and dissemination

The Swedish Ethical Review Authority approved the study protocol on 27 September 2023 (reference: 2023-05247-01). The recruitment started on 19 February 2024 and the first part is planned to end on 31 December 2027. Study results will be disseminated by means of scientific publications, presentations at conferences, popular articles, interviews, chronicles, and books. News items will be spread via social media, websites, and newsletters.

Trial registration number ISRCTN93856392 (www.isrctn.com)

Strengths and limitations of this study

- The feasibility of this randomized-controlled trial design has previously been piloted.
- This study will be carried out by a multi-disciplinary research team with strong support from patient and public involvement.
- This study aims to investigate long-term health effects of a wilderness program.
- This study will include a heterogeneous cancer study population.
- Comparison to no program participation or a wait-list control, will not be included in this randomized-controlled trial.

For peer review only

For peer review only - http://bmjopen.bmj.com/site/about/guidelines.xhtml

INTRODUCTION

Adolescent and young adults (AYAs) affected by cancer, either by a childhood or AYA cancer diagnosis, are a growing but understudied population [1]. Due to cancer or its treatment, many AYAs experience at least one late effect such as psychosocial problems, concentration/memory difficulties and fatigue that negatively impact their quality of life [2]. Furthermore, they suffer disproportionately from chronic diseases and have excess risk for early mortality compared to non-cancer populations [3]. It appears that AYAs affected by cancer receive little follow-up care that appropriately address these late effects [2]. Consequently, different self-management strategies have evolved by which they are trying to cope with late effects. These include pushing themselves physically and mentally to master everyday activities, balancing social activities and rest, and recuperating and regaining energy [4]. Other coping strategies reported are seeking distraction, support from family and friends, yoga or meditation, or changes in lifestyle such as increased physical activity and healthy diets [5]. Although AYAs affected by cancer acknowledge the importance of these self-management strategies in dealing with late effects of cancer or the cancer treatment itself, it remains an ongoing challenge [5]. Therefore, it is a high priority to find effective interventions that support the self-efficacy of AYA impacted by cancer, thereby minimizing the negative impact of cancer-related late effects and improving their quality of life [5].

Physical activity-based interventions have been shown to increase physical activity and reduce fatigue in childhood cancer survivors [6-8]. However, results must be interpreted with caution due to the small sample sizes and large heterogeneity among the reviewed studies [7]. Physical activity-based interventions have also been reported to increase the quality of life of AYA cancer survivors, however, with marginal significance [9]. It appears to be difficult for AYAs affected by cancer to return to or maintain adequate levels of physical activity, and they seem to make little use of existing supporting physical activity services within the

For peer review only - http://bmjopen.bmj.com/site/about/guidelines.xhtml

community [10]. A number of barriers exist that make it more difficult to engage in physical activity including self-reported fears about health, low baseline activity levels and/or fitness, lack of motivation, cancer-related pain or fatigue, age at diagnosis, anxiety about health, and lack of advice from a physician [11, 12].

Another promising intervention in support of the self-management of cancer survivors are nature-based interventions. Those affected by cancer have reported that engaging with nature is important for their health and well-being [13-15]. Research from different fields indicate that nature-based interventions may decrease anxiety, depression, stress, tension, fatigue, pain, and improve sleep in those affected by cancer [16]. Nature may provide a safe space and motivational context for cancer patients that supports relaxation, recovery, physical activity and may ignite reflection, inspiration, personal growth, and supportive relationships [14, 17, 18]. Furthermore, it has been demonstrated that spending time in nature leads to more frequent or longer stays in nature [19].

To gain insight into possible beneficial effects of nature-based interventions specifically for AYAs affected by cancer, we first performed a scoping review on the topic [20]. Nature-based interventions such as adventure and wilderness programs were found to increase self-efficacy, self-esteem, self-confidence, physical activity, and social support among AYAs affected by cancer. However, it was found that randomized controlled trials (RCTs) on effectiveness of nature-based interventions were lacking. Other gaps identified were the lack of studies on the long-term effects (> 3 months) of nature-based interventions, and lack of data on the safety of these interventions [20]. Therefore, in 2021-2022, we performed a pilot study to investigate whether it would be feasible to perform a RCT design on a nature-based intervention [21]. For this pilot study we developed a Wilderness program for AYAs (WAYA program) affected by cancer. The WAYA program is grounded in Næss's ecosophy [22] and the Positive Health Model [23]. A detailed description and schematic outline of the WAYA

For peer review only

program theory and content has previously been published [24]. We demonstrated that it was feasible and safe to conduct a pilot RCT [25]. None of the 42 AYAs that participated in the interventions dropped-out from the study, and adherence to program interventions and data completeness after one-year follow-up was high. The WAYA program was found to be just as safe for AYAs affected by cancer as a hotel stay. The relative frequency of reported adverse effects (AE) was similar in both study arms (Relative Risk: 1.0, 95% Confidence Interval 0.8-1.3), and all AEs were non-serious, mild, or moderate of severity, and resolved during the study period [25]. Furthermore, the WAYA program appeared to be acceptable, developmentally appropriate and of interest to AYA affected by cancer [24, 25]. Based on these observations we recommended for a future larger RCT to: 1) Apply additional recruitment strategies for participants, 2) To stratify for the inclusion of cancer survivorship (childhood cancer versus AYA cancer), 3) To optimize the content of the WAYA program and 4) To select health-related outcomes based on the lived experiences of participants in the WAYA program [26].

The aim of the present study is to perform a full-scale multi-centre RCT (WAYA-2 study) that investigates the effectiveness and safety of a wilderness program on the mental and physical well-being of AYAs affected by cancer. The WAYA program and WAYA-2 study protocol have been optimized according to the findings and recommendations from the pilot study as described above.

For peer review only - http://bmjopen.bmj.com/site/about/guidelines.xhtml

METHODS AND ANALYSIS

Study guidance and design

The WAYA-2 study protocol is developed in line with the Standard Protocol Items: Recommendations for Interventional Trials (SPIRIT) 2013 guidance for protocols of clinical trials and the SPIRIT 33-item checklist [27]. The WAYA-2 study will be conducted in accordance with the guidelines of the Declaration of Helsinki, and results will be reported according to the Consolidated Standards of Reporting Trials (CONSORT), 25-item checklist and flow diagram [28]. This is a multi-centre RCT in Norway and Sweden with a two-armed parallel design using individual randomization. A quantitative RCT design will allow the testing of active aspects of a complex wilderness intervention including physical activity, physical challenges, experiential activities, being in nature and other reflective practices. A hotel stay is chosen as a comparison to control for other possible effective factors such as attention, group support and getting out of their regular environment.

Research questions

The following research questions will be addressed:

Does a wilderness program, including a wilderness expedition and basecamp stay:

1. Increase psychological well-being of AYAs affected by cancer compared to a control activity?

2. Increase nature connectedness of AYAs affected by cancer compared to a control activity?

3. Improve health-related quality of life of AYAs affected by cancer compared to a control activity?

4. Increase physical activity level of AYAs affected by cancer compared to a control activity?

5. Is a wilderness program equally safe for AYAs affected by cancer as a hotel stay (control activity)?

For peer review only - http://bmjopen.bmj.com/site/about/guidelines.xhtml

Participants and inclusion criteria

Participants are adolescents and young adults aged 16-39 years, that have been diagnosed with any type of cancer during childhood, adolescence, or young adulthood. Another inclusion criterium is the ability to walk 2 km a day (walking aids or assistance from another person is permitted). Participants with various disabilities such as mobility, vision and hearing impairments, balance problems and special treatment or dietary needs can be included. Prior experience with outdoor activities is not required for study participation.

Exclusion criteria are:

- Cancer treatment that imposes unwanted risks for AYAs upon program participation, as evaluated by their treating physician/oncologist.
- A medical condition of AYAs that prevents safe travel to, or participation in the program, as evaluated by their treating physician/oncologist.
- Not willing to be randomly assigned to program interventions.
- Previous participation in the WAYA-1 study.

Following the recommendations of AYA cancer experts in our advisory board, we will deliberately refer to participants in the WAYA-2 study as AYAs affected by cancer, rather than childhood and AYA cancer survivors.

Intervention group: Wilderness program

The development of the Wilderness program for Adolescent and Young Adults (WAYA program) affected by cancer has previously been published [24]. The WAYA program aims to increase physical activity, self-confidence, self-efficacy and self-care, to support personal growth and building meaningful relationships, and to provide an enjoyable and safe stay in nature. Nature in the WAYA program has both a contextual and an active role in support of the health and well-being of AYAs affected by cancer, wherein their health is regarded to be

For peer review only - http://bmjopen.bmj.com/site/about/guidelines.xhtml

intrinsically interwoven in a bi-directional fashion with the health and well-being of nature [29]. Participants are given the opportunity to experience a diversity of natural landscapes, and specific nature characteristics are actively incorporated in program activities. Activities in the program include hiking, sea-kayaking, backpacking, rock climbing, camping. The program also includes (nature) reflective practices such as mindfulness, meditation, and forest bathing [24]. Outdoor skills taught include mapping/compass/orienting, trail cooking, safety training, equipment planning, foraging, fishing, bush-craft skills and leave no trace. The wilderness program consists of three intervention parts: 1) An eight-day intervention that includes two days for traveling and physical testing and a six-day wilderness expedition; 2) A three-month in-between period where participants are contacted once every month online to coach them to engage in their own outdoor activities; 3) A four-day intervention including a two-day base camp program. Both the expedition and basecamp program will take place in nature settings around the High Coast of Sweden or in Southern Norway. The group size will be 10-12 participants, and the facilitator-participant ratio will be 3:10. The six-day wilderness expedition will be logistically supported by a team of 5-7 volunteers.

Control group: Hotel stay

The hotel stay will be for eight-days at a Spa Hotel in Sweden or Norway, including two days for traveling and physical testing and a six-day hotel stay, followed by a second four-day stay at the same hotel (including two travel days) three months later. Similar to the wilderness intervention, during the three-month in-between period, participants will be contacted once every month to ask how they are doing. The content of the hotel stay includes the following activities: Spa facilities, museum visits, watching tv/movies, playing games/gaming, shopping, and fine dining. Participants will engage in organized group activities, but this does not include any nature-based activities. The group size is 10-12 participants, and the facilitator-participant ratio will be 3:10.

For peer review only - http://bmjopen.bmj.com/site/about/guidelines.xhtml

Program facilitators

In Sweden and Norway, we will work with professional teams of outdoor instructors and facilitators that have previous experience in wilderness programs with young cancer survivors or other vulnerable youth populations. Facilitators of the WAYA program have competence in one or more of the following areas: outdoor life, survival, nature/wilderness guiding, kayaking, climbing, nursing, first aid, (youth/group) counselling/supervising, mindfulness, mind-body techniques, and data collection for research purposes [24]. At least two facilitators of the WAYA program are licensed health professionals (nurses, psychologists) and at least one facilitator of the WAYA program and hotel stay is an AYA affected by cancer. For program intervention fidelity, referring to the degree to which the WAYA program is performed in a similar manner in Sweden and Norway, two members of the Norwegian outdoor team will join the Swedish outdoor team for the first wilderness intervention in Sweden, and vice versa. For supervision of participants in the hotel stay, facilitators will have competence in guiding/supervising of groups, first aid, and research methodology for data collection and safety monitoring.

Outcomes

The selection of outcomes is driven by the aims of the WAYA program and recently published qualitative study results of the WAYA program [26]. The aims of the WAYA program and underlying process to support self-realization via nature connectedness is hypothesized to contribute to a chain of actions that impacts the six domains of Positive Health [25]. The WAYA program is a complex intervention and may potentially impact on multiple factors. Therefore, in line with guidance and instructions for RCTs on complex interventions, more than one primary outcome is chosen in relation to the research questions [30].

For peer review only - http://bmjopen.bmj.com/site/about/guidelines.xhtml

Primary outcomes (confirmatory)

1. Changes in psychological well-being of AYAs affected by cancer over time, measured by the Psychological Well-being Scale (PWS) [31]. This scale consists of six domains: Self-acceptance, positive relations with others, autonomy, environmental mastery, purpose in life, and personal growth. Together, these dimensions can contribute to the assessment of a person's level of positive functioning and well-being. This instrument is translated and validated in the Swedish context.

Rationale: Significant and relevant changes in the PWS have been demonstrated for complex interventions including mindfulness-based programs [32]. The PWS was not included as an outcome measure in our previous pilot study [1] but based on the findings from the qualitative study of the WAYA program we have selected PWS as a primary outcome measure [26]. For the qualitative analysis of the pilot study, participants in the wilderness program were interviewed at 3 months after start of the program to explore the impact of the program on their health and well-being. Themes that arose through the inductive content analysis related to self-confidence, self-acceptance, meaningful relationships, and personal growth. These themes are also reflected within the domains of the PWS providing additional richness to the data [31].

2. Changes in nature connectedness of AYAs affected by cancer over time, measured via the Nature Relatedness Scale (NRS) [33]. The NRS is a 21-item scale that measures the 'affective, cognitive, and physical relationship individuals' have with the natural world. For the purpose of the previous pilot study [25], the NRS was translated and validated in the Swedish language (results not published).

Rationale: In line with guidance and instructions for RCTs on complex interventions [30], nature connectedness is hypothesized to be the underlying mechanism (intermediate outcome)

For peer review only - http://bmjopen.bmj.com/site/about/guidelines.xhtml

along the theorized impact path towards eudaemonic (psychological) well-being. A significant increase in NRS has previously been demonstrated in childhood and AYA cancer survivors after participation in the WAYA program compared to the hotel stay [25].

Secondary outcomes (exploratory)

Secondary outcomes explore possible changes in quality of life, physical activity, and fitness:

1. Changes in quality of life of AYAs affected by cancer over time, measured by the Minneapolis Manchester Quality of Life instrument (MMQL) [34]. The MMQL is a quality-of-life questionnaire specifically designed for cancer survivors and consists of seven quality of life domains: physical functioning, cognitive functioning, psychological functioning, body image, social functioning, intimate relations, and outlook on life. The instrument is translated and validated in the Swedish context.
2. Changes in light, and moderate-to-vigorous intensity physical activity will be measured objectively using ActiGraph [35].
3. Changes in maximal oxygen consumption (VO2max; ml/kg/min) will be estimated by means of the Ekblom Bak cycle ergometer test [36].
4. Blood Pressure and Heart Rate (BP/HR) will be assessed with an electronic monitor in a seated position after a 5-min rest. Two measurements will be performed, and the lowest value will be registered.
5. Body mass index (BMI) will be measured non-fasted and in light clothing to the nearest 0.1 kg by a digital scale. Height will be measured to the nearest 1 mm by a stadiometer. BMI (kg/m2) will be calculated.

Considering the large similarity between the Swedish and Norwegian language, the validated Swedish primary outcome scales (PWS, NRS, MMQL) will only be translated to the Norwegian language and back translated for quality control.

For peer review only - http://bmjopen.bmj.com/site/about/guidelines.xhtml

Safety-related outcomes

Occurrence of (serious) adverse effects during the interventions are reported and documented in the field diaries. Adverse effects will be coded according to the Medical Dictionary for Regulatory Activities (MedDRA) [37] and analyzed using preferred terms and allocation to system organ class. Safety of the programs will be evaluated by analyzing the number, seriousness, intensity, and types of adverse effects that are evaluated to be certain, probable/likely, or possibly related to the study programs. Equal safety between the wilderness program and hotel stay is defined as no significant differences in the risk of having serious adverse effects upon wilderness versus hotel stay participation.

Data collection

Table 1 below gives an overview of timepoints for data collection in the proposed study. The first part of this study has a follow-up time of one year. Additional to primary and secondary outcomes, other data will be collected for program comparison, evaluation, study quality and reporting (see Table 1). Lifestyle changes include diet, sleep, stress, physical activity (type, frequency/length), smoking, nicotine use, alcohol, caffeine, and outdoor/nature activities (type, frequency/length). Supportive relationships concern the experienced relationships with others affected by cancer (scale 1-5, not at all to very much). Self-efficacy outdoors concerns the confidence to spend time in nature/outdoors and their outdoor skills (scale 1-5, not at all to very much).

Table 1. Timeline of measurements in the study

**Measurements**	**Screening**	**Randomization**	**Intervention 1**		**Three-months at home**		**Intervention 2**	**One year**
	Contact 1	Contact 2	Day 1 **T=0***	Day 8* **T=1***	Contact 3	Contact 4	Month 3 **T=2***	Year 1 **T=3**
**Primary outcomes**								
PWS			X	X			X	X

For peer review only - http://bmjopen.bmj.com/site/about/guidelines.xhtml

NRS			X	X			X	X
**Secondary outcomes**								
MMQL			X	X			X	X
Physical activity			X	X			X	X
VO2max			X				X	
Blood pressure			X				X	
Heart rate			X				X	
Body mass index			X				X	
Adverse effects				X	X	X	X	X
**Other data**								
Willingness	X							
Preference	X							
Expectations	X							
Adherence				X			X	
Effectiveness		X			X	X		X
Demographic data	X							
Medical History		X						
Medication use		X			X	X		X
Other therapies/ self-care		X			X	X		X
Lifestyle changes		X			X	X		X
Supportive relationships		X			X	X		X
Self-efficacy outdoors		X			X	X		X
Outdoor activities		X			X	X		X
Physical activities		X			X	X		X

*To avoid possible effects of intervention-related expectations and anxiety and return-euphoria, all self-reported questionnaires will be filled in by participants 2-3 weeks before the start of the first intervention, 2-3 weeks after intervention, and 2-3 weeks after the second intervention. PWS: Psychological Well-being Scale, NRS: Nature Relatedness Scale, MMQL: Minneapolis Manchester Quality of Life instrument, VO2max: Maximal oxygen consumption. Willingness: participants' willingness to be randomized, Preference: participants' intervention preference, Expectations: participants' intervention expectations, Adherence: participants' intervention adherence, Effectiveness: participants' expected intervention effectiveness.

For peer review only

For peer review only - http://bmjopen.bmj.com/site/about/guidelines.xhtml

Sample size

The current study aims to recruit a total of 150 participants that will be randomized into two groups (*n* = 75 per group). The power calculation is based on a two-group linear mixed effects model for repeated measures data with attrition [38] and provides 80% power to detect a medium-sized between-group difference (*SMD* = 0.60). In the sample size estimation, we account for an attrition rate of 34.5% before the start of the intervention as reported in the previous pilot study [25] and a low attrition rate (10%) during the intervention. The power calculation is based on a model with four repeated measurement points, a first-order autoregressive (AR) correlation structure, an initial repeated measures correlation of 0.60 (with decreasing magnitude of the repeated measures correlations with increasing distance between time points), an alpha level of .05, and a group allocation of 1:1.

The effect size estimates for the primary outcomes are based on a meta-analytic estimate [32] of a standardized between-group difference of 0.69 (Hedges' *g*) for interventions to enhance eudaemonic psychological well-being, and findings from a pilot study indicating a significant between-group difference in NRS between the wilderness program and hotel stay (Cohen's *d* = 0.63) [25].

Recruitment

Participants will be recruited among members of the Swedish cancer organizations Ung Cancer and the Childhood Cancer Fund, members of the Norwegian cancer organization Ung Kreft, and through oncology departments of hospitals in Norway and Sweden. In all news items, information and brochures, AYAs are directed to the website of the WAYA-2 study where they can register their interest for possible participation in the study [39].

Randomization and concealment

Participants will be randomized equally (1:1) to the wilderness program or hotel stay according to a randomization list as generated by a Random Allocation Software Program

For peer review only - http://bmjopen.bmj.com/site/about/guidelines.xhtml

For peer review only

[40] using a random block size of two to guarantee a balanced allocation. Participants will be stratified according to two age groups (16-30, 31-39 years), binary gender categorizations (male/female), and cancer survivorship (childhood cancer: age 0-14 years versus AYA cancer: age 15-39 years) to achieve equal distribution among the two groups. Per stratification and per country (Norway, Sweden), separate randomization lists will be generated. AYAs affected by cancer that have registered their interest in the study via the website will receive a screening number based on the date of registration entry. Eligible participants will be randomized and assigned to the wilderness program or hotel stay based on the fixed order of the screening numbers by a researcher that is not involved in the screening and intake of study participants. Allocation to program intervention will be concealed by the complexity of the randomization procedure involving a group of ten or more eligible participants at a time and randomizations over eight different strata during the period of one year.

Blinding

Upon screening, participants will be informed that two possibly effective programs will be investigated. To reduce expectation bias, it will not be made known to participants whether the wilderness program is hypothesized to be better than a hotel stay (single-blinded). Statistical analysis will be performed by a statistician that is blind to program allocation.

Data management and confidentiality

Study data of each participant will be handled in a case report form (CRF). Self-reported demographic data, medical data (cancer diagnosis, cancer treatment, medical history etc), and other relevant intervention-related data (preference, expectations, effectiveness etc) will also be documented in the CRF. Participants are allowed to continue all their medication, therapies and/or dietary and lifestyle measures during the study. Any changes in medication, therapies, dietary and lifestyle measures over time will be documented in the CRF. CRF data will be anonymised for analysis by assigning each participant an unidentifiable three-digit screening

For peer review only - http://bmjopen.bmj.com/site/about/guidelines.xhtml

number at the time of enrolment and additional three-digit study number upon randomization. The CRF, SPSS dataset and other participant-related documentation will be stored on a secure server at Mid Sweden University or Agder University and can only be accessed with a password by research team members that are authorized. To guarantee data quality, independent checks during data collection and data entry will be performed by another researcher than the one collecting or entering the data in the database. A data monitoring committee will not be established because of the nature of the study (health-promotion intervention with low risk). The collected data will be saved for at least ten years after the end of the study.

Data analysis

Chi-square statistics, independent samples t-tests, and analysis of variance (ANOVA) will be used to calculate group differences in socio-demographic variables and cancer characteristics at baseline. The Alpha level will be set to 0.05. The primary analysis will investigate changes in the total scores of the PWS and NRS over time from baseline to 1-year follow-up in the intervention group compared to the control group. Missing data will be imputed by means of multiple imputation or by using full-information maximum likelihood estimation [41]. The analysis of the primary outcome will be based on a linear mixed effects model and will be fitted to test intervention effects up to 1 year. Adjustments will be made for age, gender, age at cancer diagnoses, and study site. Time, intervention arm, interaction between time and intervention arm, age, gender, age at cancer diagnoses, and study site are included as fixed effects, and participant is included as random effect in the model. Statistical analyses will be on the intention-to-treat (ITT) principle (all participants that are randomized in the study). As sensitivity analyses, the per-protocol data set will be analysed including only participants that have participated for at least one day in the wilderness or hotel stay. Secondary outcomes will also be analysed using linear mixed effects models.

For peer review only - http://bmjopen.bmj.com/site/about/guidelines.xhtml

For peer review only

Categorical data will be presented as frequencies and percentages. For continuous data, the number, mean, standard deviation, median, inter-quartile range, minimum and maximum will be calculated. A two-sided p-value <0.05 will be considered statistically significant.

Safety analyses will be performed on the safety analysis set and include all individuals that had participated in one of the program interventions for at least one day. Relative Risk (RR) with 95% Confidence Intervals (CI) will be calculated to indicate the risk of having an adverse effect adjusted to days of exposure in program interventions. Pearson's chi-squared test will be used to calculate differences in adverse effects severity between the two study arms. A detailed description of the planned statistical analyses will be provided in the Statistical Analysis Plan (SAP), which will be finalized prior to database closure and data analysis. All statistical analysis will be performed using Stata version 18.0.

Protocol amendments

Possible changes to this protocol, and reasons for these changes, will be documented in the trial register (www.isrctn.com), and if needed submitted to the Swedish and Norwegian Ethical Review Authority for approval. The registered study protocol (ISRCTN93856392) includes all items on the minimum standard check list of items of the World Health Organization [42].

Patient and Public Involvement

User involvement and collaboration with the wider community was established during our previous WAYA-1 study and will be continued in the present study. Three AYAs affected by cancer are in the advisory board of the WAYA-2 study. For all wilderness and hotel stay interventions, at least one member of the facilitating team will be an AYA affected by cancer. The WAYA-2 study design and recruitment strategies have been developed in close collaboration with AYAs affected by cancer, the Swedish cancer organizations for childhood

For peer review only - http://bmjopen.bmj.com/site/about/guidelines.xhtml

and young cancer, researchers from four different countries (Netherlands, Norway, Sweden, USA), and health care professionals from the region Västernorrland in Sweden. The content of the WAYA-2 program and hotel stay has been developed in close collaboration with the Swedish survival guilt, other outdoor instructors/nature guides, and the Swedish cancer organization and optimized through feedback from previous participants in the WAYA-1 study. All parties as mentioned above will be involved in interpretation and dissemination of study results.

Ethics and dissemination

Participants are informed both orally and in writing that participation in the WAYA-2 study is voluntary. It is made clear that participation in all study-related activities is voluntary, that they can decline participation in any activity without explanation, and that they can withdraw at any time from the program without stating a reason. Participants are further informed about the purpose of the study, that data will be handled and later published and presented in a confidential way. Informed consent will be obtained from all participants in writing prior to the start of the study and send to the researcher responsible for screening and intake (see Supplemental Material 1 for an example of the participant consent form). In Norway and Sweden, participants from age 16 can provide individual informed consent without permission/signature from parents/guardians. Participation in the WAYA program is without costs for participants.

The WAYA-2 study will be performed in line with the instructions and precautions as described in the study safety plan [24]. A physician with emergency medicine expertise will be available by telephone in case of emergency. Adequate insurance for all participants and facilitators in the study will be provided by Mid Sweden University or Agder University. The study protocol was approved by the Swedish Ethical Review Authority on 27 September 2023 (reference: 2023-05247-01). The study application to the Norwegian Ethical Review

For peer review only - http://bmjopen.bmj.com/site/about/guidelines.xhtml

Authority is pending (reference: 694114). Information letters and consent forms will be accessible upon request.

We aim for a wide communication and dissemination of research results through close collaboration with national and international research partners and the wider communities in the respective countries. Results of the study will be presented to the participants. It is aimed to publish at least three scientific (peer-reviewed) articles and to present the results at (scientific) national and international conferences. Popular articles with study results will be disseminated in the form of interviews, books, and chronicles. News items will also be spread via social media (Facebook, Twitter), websites and newsletters of collaborating partners and organizations and of the funding bodies.

Timeline of the study

The timeline of the study is depicted in Figure 1. The first participants were enrolled in the study on 19 February 2024. The first interventions in Sweden will be carried out during the summer of 2024. The interventions in Norway will take place in the summer of 2025. Since participants are followed for one year after the start of the intervention, data collection will end in July 2026. Data analyses are planned to start in December 2026 and reporting is planned to end in December 2027. It is the aim of this study that participants will be followed up for ten years after the study start (two-, five- and ten-year follow-up) to investigate possible long-term changes in psychological well-being, health related quality of life and physical activity. Ethical approval and finance for the long-term follow-up of the WAYA-2 study will be sought in 2025.

For peer review only - http://bmjopen.bmj.com/site/about/guidelines.xhtml

DISCUSSION

AYAs affected by cancer are an understudied and vulnerable group and effective interventions are needed to ensure that they are able to cope with late effects of cancer, its treatment, and achieve good quality of life. To the best of our knowledge, worldwide this study will be the first full-scale RCT that investigates the effectiveness of a wilderness program for AYAs affected by cancer, and the first wilderness program in Scandinavia that is developed and adapted to their needs. The WAYA program in this study is specifically designed to offer a supervised wilderness program to AYAs with a wide range of disabilities [24].

This study aims to demonstrate the evidence base for a wilderness program intending to impact the psychological well-being of AYAs affected by cancer. Expected short-term impact is that the wilderness program may support their self-efficacy, self-confidence, and provide them with the competence to develop and maintain their own outdoor practice. The long-term impact of the wilderness program may decrease depression, anxiety, and stress, and increase quality of life. In the development of the previous pilot RCT design, considerable effort was invested to define an appropriate control group activity [21]. A hotel stay was chosen to control for attention from facilitators, group support, and being away from their home environment. Although it might be expected that relaxation and connecting with other AYAs offer positive experiences and time to recharge, it is expected that these may have less impact on their health and well-being in the long-term [43, 44]. Based on previous experiences with the feasibility of a planned RCT in Norway [45], it was decided not to include a "care as usual" or wait-list control arm in the WAYA-2 study.

This study is expected to fill a gap in the literature by providing valuable insights and needed evidence on the role of nature in the support of the health and well-being of such a vulnerable

For peer review only - http://bmjopen.bmj.com/site/about/guidelines.xhtml

and understudied population. This will inform AYAs affected by cancer, their families, outdoor instructors, clinicians, and other researchers.

One of the expected challenges of the present study is to recruit the needed number of participants in the study within the set timeframe. We previously published that the eligibility (87,3%) and enrolment rate (95.2%) of participants in the WAYA-1 study were high [25]. To randomize 150 eligible individuals into the WAYA-2 study, we therefore need to recruit at least 180 AYAs affected by cancer to register their interest to participate in the WAYA-2 study. In our previous pilot study we only recruited among members of Swedish cancer organizations Ung Cancer and the Childhood Cancer Fund, and the recruitment rate was low (3.6%) [25]. However, the pilot study was performed during the COVID-19 pandemic where recommendations with respect to travel restrictions were in place. It is therefore expected a higher recruitment rate in the WAYA-2 study. Furthermore, we will now also recruit participants in Norway and additional recruitment strategies will be developed with support of healthcare professionals and brochures in waiting rooms of oncology departments of hospitals in Norway and Sweden.

Another potential challenge of the WAYA-2 study is the inclusion of a heterogenous study population (all cancer diagnosis and all cancer treatment stages) with a wide age range (16-39 years). Program activities and study outcomes may be affected by participants being at very different stages of their cancer trajectory and/or lives. The inclusion of participants with a large span was investigated in detail in the previous pilot study and results have been published [24]. It appeared that the wide age range and different stages in the cancer process was perceived to be an advantage (rather than a disadvantage), since it positively influenced group bonding and learning process, according to facilitators and participants.

For peer review only

For peer review only - http://bmjopen.bmj.com/site/about/guidelines.xhtml

This study involves a unique cross-sectional international (Norway, Sweden, USA) and multidisciplinary collaboration between academic researchers, clinicians, outdoor instructors, public health professionals, patient organizations, and non-profit organizations in the outdoor sector. Members of the research team have expertise in clinical trials, epidemiology, nature-based therapies, nursing, oncology, psycho-oncology, public health, social sciences, sport sciences, wilderness therapy, and have previously published on the topic [20, 21, 24-26, 45-49]. It is expected that such a collaboration is needed to tackle the complexity of this multi-component intervention for AYAs affected by cancer.

STUDY STATUS

First participants were enrolled in the study on 19 February 2024. The estimated end of the first part of the study (one year follow-up) is planned on 31 December 2027.

ACKNOWLEDGEMENTS

The authors thank Jens Annamatz Ranvide, Susanne Johansson, and Elvira Rolandsdotter for their valuable comments, advice, and contributions to the WAYA-2 study. Sandra Häggander of Ung Cancer Sweden, Sara Hellberg and Bea Eriksson from the Swedish Childhood Cancer Fund (Maxa livet) are thanked for their constructive input and feedback related to the performance of the study.

AUTHOR CONTRIBUTIONS

EAL, MCJ, and MJ conceived the idea for the study. MJ is the principal investigator in Sweden and SB is the principal investigator in Norway. MCJ will coordinate the study in Sweden and CRF will coordinate the study in Norway. The WAYA-2 study is designed by all co-authors. MCJ drafted the first version of the protocol and manuscript. AS will perform the statistical analyses. AS, CRF, DEV, EAL, MJ and SB critically revised the study protocol and manuscript. All authors approved of the manuscript. Regarding publication of results,

For peer review only

authorship eligibility guidelines of the respective peer-reviewed scientific journals will be followed.

FUNDING STATEMENT

Research grants have been obtained from Ekhagastiftelsen (grant no 2023-29) and Sjöbergstiftelsen (grant no 2024-01-04:10) in Sweden. Funders have no possibility to influence the study design, data collection, management, and analysis, study results, writing and publication of scientific articles. In-kind financial contributions to the study will be provided for by Mid Sweden University (Sundsvall, Sweden), the National Research Center in Complementary and Alternative Medicine (NAFKAM) (Tromsø, Norway), and the University of Agder (Kristiansand, Norway).

DATA AVAILABILITY STATEMENT

The final dataset from this ongoing study will become available after publication of the results upon request to the corresponding author.

COMPETING INTERESTS STATEMENT

None declared

Figure 1. Time line of the WAYA-2 study

REFERENCES

1 Murphy CC, Lupo PJ, Roth ME, et al. Disparities in Cancer Survival Among Adolescents and Young Adults: A Population-Based Study of 88 000 Patients. *J Natl Cancer Inst* 2021;**113**(8):1074-83. doi: 10.1093/jnci/djab006 [published Online First: 2021/01/24]

For peer review only - http://bmjopen.bmj.com/site/about/guidelines.xhtml

2. Mellblom AV, Kiserud CE, Rueegg CS, et al. Self-reported late effects and long-term follow-up care among 1889 long-term Norwegian Childhood, Adolescent, and Young Adult Cancer Survivors (the NOR-CAYACS study). *Support Care Cancer* 2021;**29**(6):2947-57. doi: 10.1007/s00520-020-05790-6
3. Oeffinger KC, Mertens AC, Sklar CA, et al. Chronic health conditions in adult survivors of childhood cancer. *N Engl J Med* 2006;**355**(15):1572-82. doi: 10.1056/NEJMsa060185 [published Online First: 2006/10/13]
4. Larsen MH, Larsen EH, Ruud E, et al. "I have to do things differently now, but I make it work"—Young childhood cancer survivors' experiences of self-management in everyday living. *J Cancer Surviv* 2022;**16**(4):728-40. doi: 10.1007/s11764-021-01066-y
5. Crowder SL, Sauls R, Gudenkauf LM, et al. The Lived Experience of Young Adult Cancer Survivors after Treatment: A Qualitative Study. *Nutrients* 2023;**15**(14):3145.
6. Cheung AT, Li WHC, Ho LLK, et al. Physical activity for pediatric cancer survivors: a systematic review of randomized controlled trials. *J Cancer Surviv* 2021;**15**(6):876-89. doi: 10.1007/s11764-020-00981-w [published Online First: 2021/01/04]
7. Moberg L, Fritch J, Westmark D, et al. Effect of physical activity on fatigue in childhood cancer survivors: a systematic review. *Support Care Cancer* 2022;**30**(8):6441-49. doi: 10.1007/s00520-022-06960-4
8. Wogksch MD, Goodenough CG, Finch ER, et al. Physical activity and fitness in childhood cancer survivors: A scoping review. *Aging and Cancer* 2021;**2**(4):112-28. doi: https://doi.org/10.1002/aac2.12042
9. Zhi X, Xie M, Zeng Y, et al. Effects of Exercise Intervention on Quality of Life in Adolescent and Young Adult Cancer Patients and Survivors: A Meta-Analysis. *Integr Cancer Ther* 2019;**18**:1534735419895590. doi: 10.1177/1534735419895590 [published Online First: 2019/12/18]
10. Adams SC, Petrella A, Sabiston CM, et al. Preferences for exercise and physical activity support in adolescent and young adult cancer survivors: a cross-sectional survey. *Support Care Cancer* 2021;**29**(7):4113-27. doi: 10.1007/s00520-020-05897-w
11. Cox CL, Montgomery M, Oeffinger KC, et al. Promoting physical activity in childhood cancer survivors: results from the Childhood Cancer Survivor Study. *Cancer* 2009;**115**(3):642-54. doi: 10.1002/cncr.24043 [published Online First: 2009/01/02]
12. Xu FJ ZL, Miao BH, Wang BJ, Li H, Chen Y, Wang HM,. Research progress on physical activity of childhood cancer survivors. *Chinese journal of preventive medicine* 2024;**58**(2):177-82.

For peer review only

13. Ahmadi F, Ahmadi N. Nature as the Most Important Coping Strategy Among Cancer Patients: A Swedish Survey. *J Relig Health* 2015;**54**(4):1177-90. doi: 10.1007/s10943-013-9810-2 [published Online First: 2013/12/24]

14. Blaschke S. The role of nature in cancer patients' lives: a systematic review and qualitative meta-synthesis. *BMC Cancer* 2017;**17**(1):370. doi: 10.1186/s12885-017-3366-6 [published Online First: 2017/05/27]

15. Blaschke S, O'Callaghan CC, Schofield P. Cancer Patients' Recommendations for Nature-Based Design and Engagement in Oncology Contexts: Qualitative Research. *HERD: Health Environments Research & Design Journal* 2017;**11**(2):45-55. doi: 10.1177/1937586717737813

16. Timko Olson ER, Olson AA, Driscoll M, et al. Nature-Based Interventions and Exposure among Cancer Survivors: A Scoping Review. *Int J Environ Res Public Health* 2023;**20**(3) doi: 10.3390/ijerph20032376 [published Online First: 2023/02/12]

17. Lesser IA, Nienhuis CP, Belanger L. Active by nature: exploring cancer survivors' exercise barriers, facilitators, preferences, and psychosocial benefits of engaging in outdoor physical activity. *Support Care Cancer* 2021;**29**(7):4095-103. doi: 10.1007/s00520-020-05938-4

18. Park EY, An MY, Sung JH. A present from the forest: Focus group interviews on cancer survivors' forest experiences. *Asia Pac J Oncol Nurs* 2022;**9**(8):100105. doi: 10.1016/j.apjon.2022.100105 [published Online First: 2022/09/06]

19. de Vries S, Verheij RA, Groenewegen PP, et al. Natural Environments—Healthy Environments? An Exploratory Analysis of the Relationship between Greenspace and Health. *Environment and Planning A: Economy and Space* 2003;**35**(10):1717-31. doi: 10.1068/a35111

20. Jong M, Lown EA, Schats W, et al. A scoping review to map the concept, content, and outcome of wilderness programs for childhood cancer survivors. *PLOS ONE* 2021;**16**(1):e0243908. doi: 10.1371/journal.pone.0243908

21. Jong MC, Mulder E, Kristoffersen AE, et al. Protocol of a mixed-method randomised controlled pilot study evaluating a wilderness programme for adolescent and young adult cancer survivors: the WAYA study. *BMJ Open* 2022;**12**(5):e061502. doi: 10.1136/bmjopen-2022-061502

22. Naess A. Self-realization: An ecological approach to being in the world. *Trumpeteer* 1987;**4**:35-42.

23. Huber M, van Vliet M, Giezenberg M, et al. Towards a 'patient-centred' operationalisation of the new dynamic concept of health: a mixed methods study. *BMJ Open* 2016;**6**(1):e010091. doi: 10.1136/bmjopen-2015-010091

For peer review only - http://bmjopen.bmj.com/site/about/guidelines.xhtml

24. Jong MC, Stub T, Mulder E, et al. The Development and Acceptability of a Wilderness Programme to Support the Health and Well-Being of Adolescent and Young Adult Cancer Survivors: The WAYA Programme. Int J Environ Res Public Health 2022;19(19):12012.
25. Jong MC, Dahlqvist H, Lown EA, et al. A randomized controlled pilot study assessing feasibility and safety of a wilderness program for childhood, adolescent, and young adult cancer survivors: the WAYA study. BMC Public Health 2023;23(1):1504. doi: 10.1186/s12889-023-16408-x
26. Jong M, Stub T, Jong MC. Supporting Personal Growth in Childhood, Adolescent and Young-Adult Cancer Survivors Through Challenges in Nature — A Qualitative Study of WAYA Wilderness Programme Participation. *Global Advances in Integrative Medicine and Health* 2024;**13**:27536130241238150. doi: 10.1177/27536130241238150
27. Chan A-W, Tetzlaff JM, Gøtzsche PC, et al. SPIRIT 2013 explanation and elaboration: guidance for protocols of clinical trials. *BMJ : British Medical Journal* 2013;**346**:e7586. doi: 10.1136/bmj.e7586
28. CONSORT 2010 Statement: Updated Guidelines for Reporting Parallel Group Randomized Trials. *Ann Intern Med* 2010;**152**(11):726-32. doi: 10.7326/0003-4819-152-11-201006010-00232 %m 20335313
29. Naess A. Ecology, community and lifestyle. Cambridge: Cambridge University Press 1990.
30. Leroy JL, Frongillo EA, Kase BE, et al. Strengthening causal inference from randomised controlled trials of complex interventions. *BMJ Glob Health* 2022;**7**(6) doi: 10.1136/bmjgh-2022-008597 [published Online First: 2022/06/11]
31. Ryff CD. Happiness is everything, or is it? Explorations on the meaning of psychological well-being. *J Pers Soc Psychol* 1989;**57**(6):1069-81. doi: 10.1037/0022-3514.57.6.1069
32. van Dierendonck D, Lam H. Interventions to enhance eudaemonic psychological well-being: A meta-analytic review with Ryff's Scales of Psychological Well-being. *Applied Psychology: Health and Well-Being* 2023;**15**(2):594-610. doi: https://doi.org/10.1111/aphw.12398
33. Nisbet EK, Zelenski JM, Murphy SA. The Nature Relatedness Scale:Linking Individuals' Connection With Nature to Environmental Concern and Behavior. *Environment and Behavior* 2009;**41**(5):715-40. doi: 10.1177/0013916508318748
34. Bosworth A, Goodman EL, Wu E, et al. The Minneapolis-Manchester Quality of Life Instrument: reliability and validity of the Adult Form in cancer survivors. *Qual Life Res*

For peer review only - http://bmjopen.bmj.com/site/about/guidelines.xhtml

2018;**27**(2):321-32. doi: 10.1007/s11136-017-1671-9 [published Online First: 2017/08/05]

35. Berntsen S, Hageberg R, Aandstad A, et al. Validity of physical activity monitors in adults participating in free-living activities. *Br J Sports Med* 2010;**44**(9):657-64. doi: 10.1136/bjsm.2008.048868 [published Online First: 2008/07/17]

36. Björkman F, Ekblom-Bak E, Ekblom Ö, et al. Validity of the revised Ekblom Bak cycle ergometer test in adults. *Eur J Appl Physiol* 2016;**116**(9):1627-38. doi: 10.1007/s00421-016-3412-0 [published Online First: 2016/06/18]

37. Medical Dictionary for Regulatory Activities (MedDRA®)2007.

38. Lu K, Luo X, Chen PY. Sample size estimation for repeated measures analysis in randomized clinical trials with missing data. *Int J Biostat* 2008;**4**(1):Article 9. doi: 10.2202/1557-4679.1098 [published Online First: 2008/01/01]

39. Mid Sweden University. WAYA-2 study 2024 [Available from: https://www.miun.se/waya-anmalan accessed 1 March 2024.

40. Ltd. SE. Create a blocked randomisation list 2022 [Available from: https://www.sealedenvelope.com accessed 12 February 2024.

41. Enders C. Missing Data: An Update on the State of the Art. *Psychol Methods* 2023 doi: 10.1037/met0000563

42. WHO. WHO Trial Registration Data Set [Version 1.3.1:[Available from: https://www.who.int/clinical-trials-registry-platform/network/who-data-set accessed 29 February 2024.

43. de Bloom J, Geurts SAE, Taris TW, et al. Effects of vacation from work on health and well-being: Lots of fun, quickly gone. *Work Stress* 2010;**24**(2):196-216. doi: 10.1080/02678373.2010.493385

44. de Bloom J, Kompier M, Geurts S, et al. Do we recover from vacation? Meta-analysis of vacation effects on health and well-being. *J Occup Health* 2009;**51**(1):13-25. doi: 10.1539/joh.k8004 [published Online First: 2008/12/20]

45. Gabrielsen LE, Fernee CR, Aasen GO, et al. Why Randomized Trials Are Challenging Within Adventure Therapy Research:Lessons Learned in Norway. *J Exper Edu* 2016;**39**(1):5-14. doi: 10.1177/1053825915607535

46. Harper NJ, Fernee CR, Gabrielsen LE. Nature's Role in Outdoor Therapies: An Umbrella Review. *Int J Environ Res Public Health* 2021;**18**(10) doi: 10.3390/ijerph18105117 [published Online First: 2021/06/03]

47. Lown EA, Otto HR, Norton CL, et al. Program evaluation of a wilderness experience for adolescents facing cancer: A time in nature to heal, connect and find strength. *PLOS ONE* 2023;**18**(10):e0291856. doi: 10.1371/journal.pone.0291856

For peer review only - http://bmjopen.bmj.com/site/about/guidelines.xhtml

48. Stea TH, Jong MC, Fegran L, et al. Mapping the Concept, Content, and Outcome of Family-Based Outdoor Therapy for Children and Adolescents with Mental Health Problems: A Scoping Review. *Int J Environ Res Public Health* 2022;**19**(10):5825.

49. Victorson D, Doninger G, Victorson S, et al. Psychosocial and Biological Outcomes of Immersive, Mindfulness-Based Treks in Nature for Groups of Young Adults and Caregivers Affected by Cancer: Results from a Single Arm Program Evaluation from 2016-2021. *Int J Environ Res Public Health* 2021;**18**(23) doi: 10.3390/ijerph182312622 [published Online First: 2021/12/11]

For peer review only

For peer review only - http://bmjopen.bmj.com/site/about/guidelines.xhtml

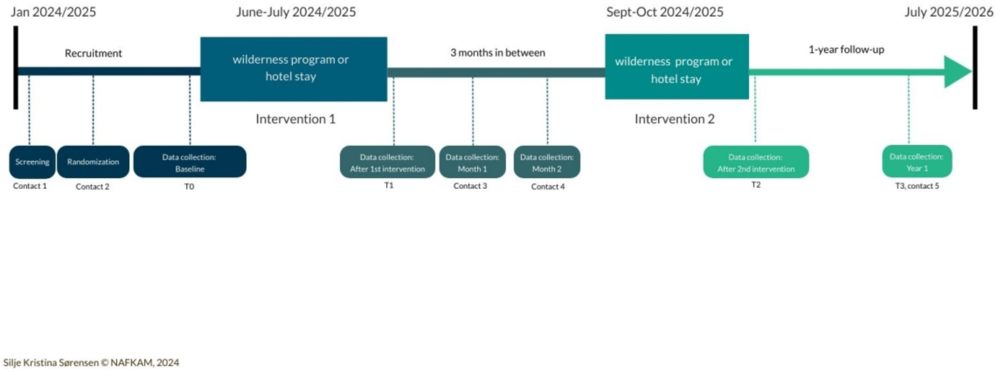

Time line of the WAYA-2 study

540x297mm (72 x 72 DPI)

En studie som undersöker säkerhet samt vilka effekter naturvistelse och vila/rekreation har på mentalt och psykiskt välmående bland unga canceröverlevare

Mittuniversitetet
MID SWEDEN UNIVERSITY
Institutionen för Hälsovetenskaper

<u>Information till deltagare</u>

En studie som undersöker säkerhet samt vilka effekter naturvistelse och vila/rekreation har på mentalt och psykiskt välmående bland unga canceröverlevare

Du har visat intresse för att om möjligt delta i den här studien.

Innan Du slutligen bestämmer Dig, är det viktigt att Du får veta mer om vad studien innebär. Av den anledningen är det viktigt att Du läser detta brev noggrant. Prata med din familj eller vänner, och om Du har frågor efter att ha läst informationsmaterialet så är Du välkommen att kontakta oss (ansvariga forskare, kontaktuppgifter i slutet av informationsbrevet).

De senaste åren har det bland människor i allmänhet samt inom forskning mer och mer uppmärksammats att vistelse i naturen kan bidra till hälsa och välmående.
Naturbaserade program ses som lovande vägar för att stötta ungdomar hälsa och välmående i allmänhet men det finns mycket litet forskning kring hur det fungerar/upplevs hos unga canceröverlevare och man vet inte säkert om de resutat som ses beror på om deltagare har en möjlighet till att komma bort från sin hemmiljö och slappna av, eller om det är just naturvistelse som ger känsla av välmående.

Vad är syftet med studien?

Studien är en så kallad randomiserad kontrollerad studie, där deltagarna lottas till att antingen delta i ett naturbaserat friluftsprogram eller ett semesterprogram. I studien kommer vi att undersöka om det finns skillnader mellan grupperna avseende förändringar i hälsa och välmående. Det kommer även att undersökas om båda programmen är lika säkra att genomföra.

Vem kan vara med?

Är du mellan 16-39 år gammal och är/har varit drabbad av cancer någon gång i ditt liv så kan du vara med.

Andra krav:

- Att du klara av att gå minst 2 km utan paus (kryckor som gånghjälpmedel går bra)
- Att du pratar och förstår antingen svenska, norska, danska eller engelska
- Att du accepterar att du kommer att bli lottad till deltagande i den ena eller andra gruppen

Du kan vara med även om du har andra fysiska eller psykiska funktionsnedsättningar, syn, hörsel etc, eller om du ej kan bära en ryggsäck – då ordnar vi med stöd/ledsagare.

Av säkerhetsskäl kan du ej vara med om du har ett sjukdomstillstånd eller nyligen genomgått cytostatikabehandling som innebär extra hög blödningsrisk eller infektionskänslighet – om det finns frågetecken kring det så måste vi rådgöra med din behandlande läkare för att avgöra om deltagande är säkert för just dig.

Hur går det till och vad innebär deltagandet

Totalt i studien kommer det att ingå 150 unga canceröverlevare 16-39 år gamla som rekryterats med hjälp av föreningarna Ung Cancer, Maxa Livet (Sverige, Ung Kreft (Norge), Ung Kræft (Danmark) bland föreningen Ung Cancers medlemmar. Hälften av dessa (75 personer) kommer att lottas till att delta i programmet som innehåller naturvistelse, och hälften (75 personer) till semestervistelse (en

For peer review only - http://bmjopen.bmj.com/site/about/guidelines.xhtml

avslappnande semestervecka på hotell med spa). Studien genomförs parallellt i Sverige och Norge, vilket innebär att deltagarna i Sverige (cirka 75) i huvudsak kommer att vara från Sverige.

Alla deltagare kommer att genomgå (enklare) fysiska tester avseende kondition, samt få besvara frågeformulär kopplat till grundläggande medicinsk bakgrund, hälsa och välmående både före, direkt efter, tre månader efter samt ett år efter genomgånget program.

Vidare kommer deltagarna även att intervjuas avseende deras upplevelser i samband med deltagande – detta sker i samband med tremånadersuppföljning samt ett år efter programmet startade.

Programmen

Det naturbaserade friluftsprogrammet

Deltagarna som blir lottade till friluftsprogrammet kommer i grupper om tio personer att få delta i en åtta dagars vandring med övernattning i tält (Höga Kusten, Ångermanland). Under veckan genomförs dagligen olika former av gruppövningar. Projektet tillhandahåller all nödvändig friluftsutrustning och mat under de åtta dagarna.

Semesterprogrammet

Deltagarna i semesterprogrammet kommer i grupper om tio personer få tillbringa en avslappnande semestervecka på ett hotell där det bland annat finns möjlighet till bad och spa faciliteter.

Tremånader uppföljning av båda grupperna

Båda grupperna följs under tre månader efter första programveckan och kommer att få besvara samma webbaserade frågeformulär som före programmet. Alla kommer efter tre månader även få komma ”tillbaks” för en uppföljningsträff, med ytterligare en kort vandring med övernattning i naturen för friluftsgruppen, samt avslappnande hotell/spaweekend för semestergruppen.

Programteam

Vid båda programmen kommer minst tre handledare/instruktörer att vara närvarande för att stötta och guida deltagarna. Bland dessa finns stor frilufts- och sjukvårdskompetens (Sjuksköterskor/psykolog/läkare) och det finns även expertkompetens inom cancervård knutet till teamet (Läkare).

Kostnadsfritt deltagande

Allt deltagande i studien är kostnadsfritt och projektet kommer att stå för resekostnader, mat och utrustning under deltagandet.

Projektet tecknar även en specifik olycksfallsförsäkring via Kammarkollogiet för alla deltagare under den tid de reser till/från event, samt under event.

Ersättning ges inte för eventuell förlorad arbetsinkomst.

Hur mycket tid tar deltagande för dig och vilka risker innebär deltagande

Om Du väljer att delta förväntar vi oss att Du under ett års lägger ner en del tid för att fylla i alla enkäter, samt även för resor till och från event (8+4 dagar).

Enkäterna är webbaserade och besvaras före programdeltagande, två veckor efter, två veckor efter tremånadersuppföljning, samt ett år efter start i studien.

Att fylla i de webbaserade enkäterna tar cirka 30 minuter per tillfälle och vid två tillfällen kommer du även att få genomföra en enklare konditionstest som tar cirka 30 minuter. Om vi upptäcker avvikande resultat i exempelvis blodtryck, så gör dig uppmärksam på det så att du kan kontakta din primärvårdsläkare för uppföljning.

For peer review only - http://bmjopen.bmj.com/site/about/guidelines.xhtml

Så vitt vi känner till så innebär deltagandet i studien inga extra risker för Dig utom för vad som normalt kan förväntas när personer vistas ute i skog och mark, möjligen liten risk för: träningsvärk, skavsår, vrickning, solexponering mm. Dock kommer teamet bistå i den mån det går till att förebygga att det uppstår.

Om det till följd av din cancersjukdom (eller behandling) finns extra risker för just dig är det viktigt att vi känner till dem och eventuellt måste rådgöra med din behandlande läkare.

Dina rättigheter

Deltagandet är **frivilligt** och Du kan själv välja att avstå deltagande när som helst, utan att ange skäl.

Dina svar och dina resultat kommer att behandlas så att inte obehöriga kan ta del av dem. Vid rapportering och publicering av forskningsresultat, (som kommer att ske i nationella och internationella vetenskapliga tidskrifter och populärvetenskapliga texter), kan inga enskilda individer bli identifierade i redovisningen.

Efter studiens avslut skickas rapporter och artiklar ut till de deltagare som önskar få dem.

Forskningshuvudman för projektet är Mittuniversitetet. Med forskningshuvudman menas den organisation som är ansvarig för projektet. Ansökan är godkänd av Etikprövningsmyndigheten, diarienummer för prövningen hos Etikprövningsmyndigheten är 2023-05247-01.
Dina svar och dina resultat kommer att behandlas så att inte obehöriga kan ta del av dem.
Ansvarig för dina personuppgifter är Mittuniversitetets dataskyddsombud. Enligt EU:s dataskyddsförordning har du rätt att kostnadsfritt få ta del av de uppgifter om dig som hanteras i projektet, och vid behov få eventuella fel rättade. Du kan också begära att uppgifter om dig raderas samt att behandlingen av dina personuppgifter begränsas. Rätten till radering och till begränsning av behandling av personuppgifter gäller dock inte när uppgifterna är nödvändiga för den aktuella forskningen. Om du vill ta del av uppgifterna ska du kontakta huvudansvarig forskare (se kontaktuppgifter nedan). Dataskyddsombud nås på 060-142 80 00 eller via e-post: dataskyddsombud@miun.se. Om du är missnöjd med hur dina personuppgifter behandlas har du rätt att ge in klagomål till Integritetsskyddsmyndigheten, som är tillsynsmyndighet.

Om du har frågor eller funderingar som du vill diskutera så är Du välkommen att ringa någon av studieansvariga

Studien är ett internationellt samarbetsprojekt med deltagande forskare från Mittuniversitetet i Sverige, Universitetet i Tromsø, Universitetet i Agder och Sørlandets Sjukhus, Kristiansand i Norge, samt från University of California San i USA. Andra samverkanspartners i projektet är: Föreningarna Ung Cancer, Maxa Livet, Ung Kreft (Norge), Ung Kræft (Danmark), Svenska Överlevnadssällskapet och Region Västernorrland.

Mats Jong

Docent i Omvårdnad, Leg Sjuksköterska
Institutionen för Hälsovetenskaper/Folkhälsa
Mittuniversitetet, Sundsvall
Tel: 010/1428966, 072/581 896 6
email: mats.jong@miun.se

Miek Jong

Docent i Hälsovetenskap
Institutionen för Hälsovetenskaper/Folkhälsa
Mittuniversitetet, Sundsvall
Tel: 010/1428967
email: miek.jong@miun.se

For peer review only - http://bmjopen.bmj.com/site/about/guidelines.xhtml

Mittuniversitetet
MID SWEDEN UNIVERSITY
Institutionen för Hälsovetenskap

Samtyckesformulär avseende deltagande i studie som undersöker säkerhet samt vilka effekter naturvistelse har på mentalt och psykiskt välmående bland unga canceröverlevare

Jag är tillfrågad om att delta i denna vetenskapliga studie

Namn:____

- Jag har läst informationsbrevet till deltagare. Jag hade möjlighet att ställa ytterligare frågor och dessa besvarades tillfredsställande, jag hade även tillräckligt med tid för att avgöra om jag ville delta eller inte, och får även behålla den skriftliga informationen.
- Jag förstår att deltagande är helt frivilligt samt att jag kan besluta mig om att avsluta mitt deltagande när som helst utan studiens gång utan att ange orsak.
- Jag medger att det insamlade data får användas för det syfte som beskrivets i informationsbrevet.
- Jag är medveten om att det insamlade materialet kommer att sparas i minst 13 år på Mittuniversitetet i Sundsvall innan det får förstöras.
- Jag samtycker till att ansvariga forskare i studien kan kontakta mig efter studiens avslutande för att höra efter hur det går för mig.

- Jag samtycker till att ansvariga forskare i studien kan kontakta mig för att tillfråga mig om deltagande i en uppföljningsstudie i framtiden.
  ¨Ja ¨Nej

- Om det finns tveksamheter avseende om min hälsosituation medger studiedeltagande samtycker jag till att informera min behandlade läkare om mitt deltagande. I detta avseende samtycker jag även till att studieansvarig forskare/forskningskoordinator får etablera kontakt med denna för att säkerställa att det inte föreligger direkta medicinska hinder för mig att delta i studien.
- Jag önskar delta i denna studie.

Studiedeltagarens namn:_________________________Personnummer:______________-_____

Underskrift:_________________________________ Datum:________________________

Jag bekräftar att jag har tydligt och uttömmande informerat ovan nämnda person om detta forskningsprojekt. Om det under studiens gång framkommer uppgifter som kan påverka personens samtycke till deltagande, så kommer jag att informera henne så fort som möjligt

Forskarens namn:______________________________

Underskrift:____________________________________ Datum:_______________________

For peer review only

SPIRIT

STANDARD PROTOCOL ITEMS: RECOMMENDATIONS FOR INTERVENTIONAL TRIALS

SPIRIT 2013 Checklist: Recommended items to address in a clinical trial protocol and related documents*

Section/item	Item No	Description	Addressed on page number
**Administrative information**			
Title	1	Descriptive title identifying the study design, population, interventions, and, if applicable, trial acronym	____1_________
Trial registration	2a	Trial identifier and registry name. If not yet registered, name of intended registry	____3_______
	2b	All items from the World Health Organization Trial Registration Data Set	____18________
Protocol version	3	Date and version identifier	____2________
Funding	4	Sources and types of financial, material, and other support	____23_______
Roles and responsibilities	5a	Names, affiliations, and roles of protocol contributors	____1, 23_____
	5b	Name and contact information for the trial sponsor	_____NA______
	5c	Role of study sponsor and funders, if any, in study design; collection, management, analysis, and interpretation of data; writing of the report; and the decision to submit the report for publication, including whether they will have ultimate authority over any of these activities	_____23_____
	5d	Composition, roles, and responsibilities of the coordinating centre, steering committee, endpoint adjudication committee, data management team, and other individuals or groups overseeing the trial, if applicable (see Item 21a for data monitoring committee)	_____NA______

For peer review only

**Introduction**			
Background and rationale	6a	Description of research question and justification for undertaking the trial, including summary of relevant studies (published and unpublished) examining benefits and harms for each intervention	____4-6, 7___
	6b	Explanation for choice of comparators	_____7, 21_____
Objectives	7	Specific objectives or hypotheses	______7_____
Trial design	8	Description of trial design including type of trial (eg, parallel group, crossover, factorial, single group), allocation ratio, and framework (eg, superiority, equivalence, noninferiority, exploratory)	______7______
**Methods: Participants, interventions, and outcomes**			
Study setting	9	Description of study settings (eg, community clinic, academic hospital) and list of countries where data will be collected. Reference to where list of study sites can be obtained	____ 7, 9__
Eligibility criteria	10	Inclusion and exclusion criteria for participants. If applicable, eligibility criteria for study centres and individuals who will perform the interventions (eg, surgeons, psychotherapists)	______8____
Interventions	11a	Interventions for each group with sufficient detail to allow replication, including how and when they will be administered	______8-9____
	11b	Criteria for discontinuing or modifying allocated interventions for a given trial participant (eg, drug dose change in response to harms, participant request, or improving/worsening disease)	______18____
	11c	Strategies to improve adherence to intervention protocols, and any procedures for monitoring adherence (eg, drug tablet return, laboratory tests)	______NA____
	11d	Relevant concomitant care and interventions that are permitted or prohibited during the trial	_____ 16____
Outcomes	12	Primary, secondary, and other outcomes, including the specific measurement variable (eg, systolic blood pressure), analysis metric (eg, change from baseline, final value, time to event), method of aggregation (eg, median, proportion), and time point for each outcome. Explanation of the clinical relevance of chosen efficacy and harm outcomes is strongly recommended	_____11-14___
Participant timeline	13	Time schedule of enrolment, interventions (including any run-ins and washouts), assessments, and visits for participants. A schematic diagram is highly recommended (see Figure)	__13-14, Figure 1

For peer review only - http://bmjopen.bmj.com/site/about/guidelines.xhtml

Sample size	14	Estimated number of participants needed to achieve study objectives and how it was determined, including clinical and statistical assumptions supporting any sample size calculations	_____15_____
Recruitment	15	Strategies for achieving adequate participant enrolment to reach target sample size	_____15, 22____
**Methods: Assignment of interventions (for controlled trials)**			
Allocation:			
Sequence generation	16a	Method of generating the allocation sequence (eg, computer-generated random numbers), and list of any factors for stratification. To reduce predictability of a random sequence, details of any planned restriction (eg, blocking) should be provided in a separate document that is unavailable to those who enrol participants or assign interventions	_____15-16____
Allocation concealment mechanism	16b	Mechanism of implementing the allocation sequence (eg, central telephone; sequentially numbered, opaque, sealed envelopes), describing any steps to conceal the sequence until interventions are assigned	_____15-16___
Implementation	16c	Who will generate the allocation sequence, who will enrol participants, and who will assign participants to interventions	_____15-16____
Blinding (masking)	17a	Who will be blinded after assignment to interventions (eg, trial participants, care providers, outcome assessors, data analysts), and how	_____15-16___
	17b	If blinded, circumstances under which unblinding is permissible, and procedure for revealing a participant's allocated intervention during the trial	______NA___
**Methods: Data collection, management, and analysis**			
Data collection methods	18a	Plans for assessment and collection of outcome, baseline, and other trial data, including any related processes to promote data quality (eg, duplicate measurements, training of assessors) and a description of study instruments (eg, questionnaires, laboratory tests) along with their reliability and validity, if known. Reference to where data collection forms can be found, if not in the protocol	_____16-17____
	18b	Plans to promote participant retention and complete follow-up, including list of any outcome data to be collected for participants who discontinue or deviate from intervention protocols	_____16-17___

For peer review only - http://bmjopen.bmj.com/site/about/guidelines.xhtml

Data management	19	Plans for data entry, coding, security, and storage, including any related processes to promote data quality (eg, double data entry; range checks for data values). Reference to where details of data management procedures can be found, if not in the protocol	____16-17____
Statistical methods	20a	Statistical methods for analysing primary and secondary outcomes. Reference to where other details of the statistical analysis plan can be found, if not in the protocol	_____17-18____
	20b	Methods for any additional analyses (eg, subgroup and adjusted analyses)	____ 17-18___
	20c	Definition of analysis population relating to protocol non-adherence (eg, as randomised analysis), and any statistical methods to handle missing data (eg, multiple imputation)	_____17-18____
**Methods: Monitoring**			
Data monitoring	21a	Composition of data monitoring committee (DMC); summary of its role and reporting structure; statement of whether it is independent from the sponsor and competing interests; and reference to where further details about its charter can be found, if not in the protocol. Alternatively, an explanation of why a DMC is not needed	_____17____
	21b	Description of any interim analyses and stopping guidelines, including who will have access to these interim results and make the final decision to terminate the trial	______NA____
Harms	22	Plans for collecting, assessing, reporting, and managing solicited and spontaneously reported adverse events and other unintended effects of trial interventions or trial conduct	______13_____
Auditing	23	Frequency and procedures for auditing trial conduct, if any, and whether the process will be independent from investigators and the sponsor	______NA____
**Ethics and dissemination**			
Research ethics approval	24	Plans for seeking research ethics committee/institutional review board (REC/IRB) approval	______19_____
Protocol amendments	25	Plans for communicating important protocol modifications (eg, changes to eligibility criteria, outcomes, analyses) to relevant parties (eg, investigators, REC/IRBs, trial participants, trial registries, journals, regulators)	______18_____

For peer review only - http://bmjopen.bmj.com/site/about/guidelines.xhtml

Consent or assent	26a	Who will obtain informed consent or assent from potential trial participants or authorised surrogates, and how (see Item 32)	_____18-19____
	26b	Additional consent provisions for collection and use of participant data and biological specimens in ancillary studies, if applicable	_______NA____
Confidentiality	27	How personal information about potential and enrolled participants will be collected, shared, and maintained in order to protect confidentiality before, during, and after the trial	_____16-17_____
Declaration of interests	28	Financial and other competing interests for principal investigators for the overall trial and each study site	_____24_____
Access to data	29	Statement of who will have access to the final trial dataset, and disclosure of contractual agreements that limit such access for investigators	_______16-17___
Ancillary and post-trial care	30	Provisions, if any, for ancillary and post-trial care, and for compensation to those who suffer harm from trial participation	_____19_____
Dissemination policy	31a	Plans for investigators and sponsor to communicate trial results to participants, healthcare professionals, the public, and other relevant groups (eg, via publication, reporting in results databases, or other data sharing arrangements), including any publication restrictions	_____19________
	31b	Authorship eligibility guidelines and any intended use of professional writers	____23_______
	31c	Plans, if any, for granting public access to the full protocol, participant-level dataset, and statistical code	___ 24________
**Appendices**			
Informed consent materials	32	Model consent form and other related documentation given to participants and authorised surrogates	_____19______
Biological specimens	33	Plans for collection, laboratory evaluation, and storage of biological specimens for genetic or molecular analysis in the current trial and for future use in ancillary studies, if applicable	_____NA______

*It is strongly recommended that this checklist be read in conjunction with the SPIRIT 2013 Explanation & Elaboration for important clarification on the items. Amendments to the protocol should be tracked and dated. The SPIRIT checklist is copyrighted by the SPIRIT Group under the Creative Commons "Attribution-NonCommercial-NoDerivs 3.0 Unported" license.

For peer review only - http://bmjopen.bmj.com/site/about/guidelines.xhtml
