# Supplementary material for: Protocol of a randomised controlled multicentre trial investigating the effectiveness and safety of a wilderness programme on the mental and physical well-being of adolescents and young adults affected by cancer: the WAYA-2 study
Source: BMJ Open. 2024 May 21;14(5):e087626. doi: 10.1136/bmjopen-2024-087626 (PMC11110565; doi:10.1136/bmjopen-2024-087626)
Supplement: Reviewer comments [file bmjopen-2024-087626.reviewer_comments.pdf]

## PEER REVIEW HISTORY

BMJ Open publishes all reviews undertaken for accepted manuscripts. Reviewers are asked to complete a checklist review form (**http://bmjopen.bmj.com/site/about/resources/checklist.pdf**)and are provided with free text boxes to elaborate on their assessment. These free text comments are reproduced below.

## ARTICLE DETAILS

| | |
|---|---|
| **TITLE (PROVISIONAL)** | Protocol of a randomized controlled multi-centre trial investigating the effectiveness and safety of a wilderness program on the mental and physical well-being of adolescents and young adults affected by cancer: the WAYA-2 study. |
| **AUTHORS** | Jong, Miek; Fernee, Carina Ribe; Stenling, Andreas; Lown, E. Anne; Berntsen, Sveinung; Victorson, David; Jong, Mats |

This article was not externally reviewed at BMJ Open. Protocols that have been independently assessed prior to submission to BMJ Open are usually fast-tracked to publication on the grounds that further substantial changes will not be possible. This independent assessment will usually be external, independent review for both a substantial grant award from a non-commercial or government funder and ethics approval.
